# Antibiotic use during influenza infection augments lung eosinophils that impair immunity against secondary bacterial pneumonia

**DOI:** 10.1172/JCI180986

**Published:** 2024-09-10

**Authors:** Marilia Sanches Santos Rizzo Zuttion, Tanyalak Parimon, Stephanie A. Bora, Changfu Yao, Katherine Lagree, Catherine A. Gao, Richard G. Wunderink, Georgios D. Kitsios, Alison Morris, Yingze Zhang, Bryan J. McVerry, Matthew E. Modes, Alberto M. Marchevsky, Barry R. Stripp, Christopher M. Soto, Ying Wang, Kimberly Merene, Silvia Cho, Blandine L. Victor, Ivan Vujkovic-Cvijin, Suman Gupta, Suzanne L. Cassel, Fayyaz S. Sutterwala, Suzanne Devkota, David M. Underhill, Peter Chen

**Affiliations:** 1 Department of Medicine,; 2 Women’s Guild Lung Institute,; 3 Department of Biomedical Sciences,; 4 Widjaja Foundation Inflammatory Bowel Disease Institute, and; 5 Karsh Division of Gastroenterology and Hepatology, Department of Medicine, Cedars-Sinai Medical Center, Los Angeles, California, USA.; 6 Division of Pulmonary and Critical Care, Department of Medicine, Northwestern University Feinberg School of Medicine, Chicago, Illinois.; 7 Division of Pulmonary, Allergy, Critical Care and Sleep Medicine and; 8 Center for Medicine and the Microbiome, University of Pittsburgh, Pittsburgh, Pennsylvania, USA.; 9 Department of Pathology and Laboratory Medicine and; 10 Human Microbiome Research Institute, Cedars-Sinai Medical Center, Los Angeles, California, USA.

**Keywords:** Infectious disease, Pulmonology, Bacterial infections, Influenza, Innate immunity

## Abstract

A leading cause of mortality after influenza infection is the development of a secondary bacterial pneumonia. In the absence of a bacterial superinfection, prescribing antibacterial therapies is not indicated but has become a common clinical practice for those presenting with a respiratory viral illness. In a murine model, we found that antibiotic use during influenza infection impaired the lung innate immunologic defenses toward a secondary challenge with methicillin-resistant *Staphylococcus aureus* (MRSA). Antibiotics augment lung eosinophils, which have inhibitory effects on macrophage function through the release of major basic protein. Moreover, we demonstrated that antibiotic treatment during influenza infection caused a fungal dysbiosis that drove lung eosinophilia and impaired MRSA clearance. Finally, we evaluated 3 cohorts of hospitalized patients and found that eosinophils positively correlated with antibiotic use, systemic inflammation, and worsened outcomes. Altogether, our work demonstrates a detrimental effect of antibiotic treatment during influenza infection that has harmful immunologic consequences via recruitment of eosinophils to the lungs, thereby increasing the risk of developing a secondary bacterial infection.

## Abstract 2

This manuscript demonstrates that antibiotic use during influenza infection impairs the lung defenses and increase the chance of developing a bacterial pneumonia.

## Graphical abstract

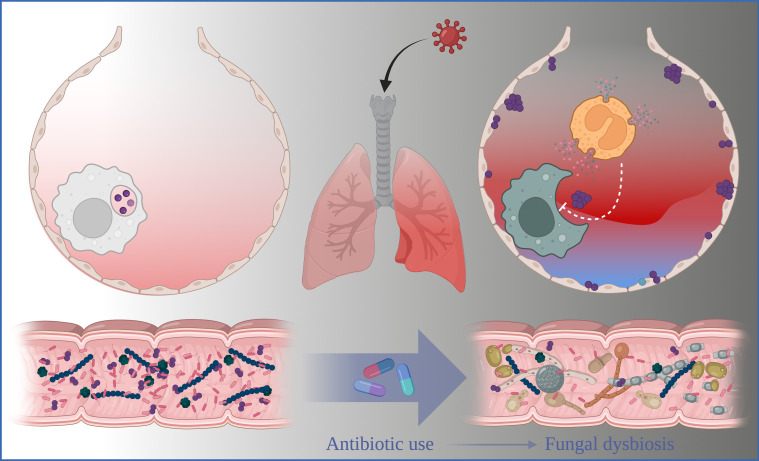

## Introduction

Influenza causes a high global burden of disease, with some estimates of attributed mortality exceeding 500,000 people annually (1, 2). The 1918–1919 influenza pandemic was particularly devastating, accounting for up to 100 million deaths worldwide (3). A major cause of mortality from influenza is development of a secondary bacterial pneumonia (4, 5). As such, the extraordinary mortality during the 1918–1919 pandemic could have been reduced if pneumococcal vaccination and antibiotics were available (6, 7). Because of the concerns for a secondary bacterial infection complicating viral pneumonia, empiric antibiotic use has become a common clinical practice (4, 5, 8–10). In fact, clinical guidelines have even suggested initiation of prophylactic antibiotics in patients presenting with a severe influenza infection (11).

During the COVID-19 pandemic, antibiotics were prescribed in nearly 75% of hospitalized COVID-19 patients, although bacterial coinfection rates were less than 10% (12). Antibiotic use during SARS-CoV-2 infection is also associated with an increase in bloodstream infections by gut bacteria (13). Therefore, empiric antibiotics in those with severe viral pneumonias will result in overprescription of these medicines where there is no clinical benefit and potential harm. Indeed, antibiotics can contribute to the emergence of multidrug-resistant bacteria and increase the risk of antibiotic-associated adverse drug events that prolong length of hospital stay (14).

We postulated that the prescription of antibiotics during influenza infection has detrimental effects that increase the risk of developing a bacterial superinfection. In a 2-hit model in which mice were first infected with influenza followed by methicillin-resistant *Staphylococcus aureus* (MRSA) infection, treatment of mice with a broad-spectrum antibiotic cocktail of vancomycin, neomycin, ampicillin, and metronidazole (VNAM) impaired lung immunity against MRSA challenge after influenza infection. VNAM induced the recruitment of eosinophils to the lungs, and we provide evidence that eosinophils can suppress macrophage clearance of MRSA through the release of eosinophil major basic protein (MBP-1). Furthermore, we demonstrated that gut fungal dysbiosis caused by antibiotics induced eosinophil recruitment, impaired MRSA clearance, and worsened lung inflammation in the 2-hit model. Human studies also corroborated our finding that eosinophil levels correlate with antibiotic use, systemic inflammation, and adverse outcomes in hospitalized patients.

## Results

### Antibiotic use during influenza infection impairs MRSA clearance and worsens lung injury.

**Figure 1:**
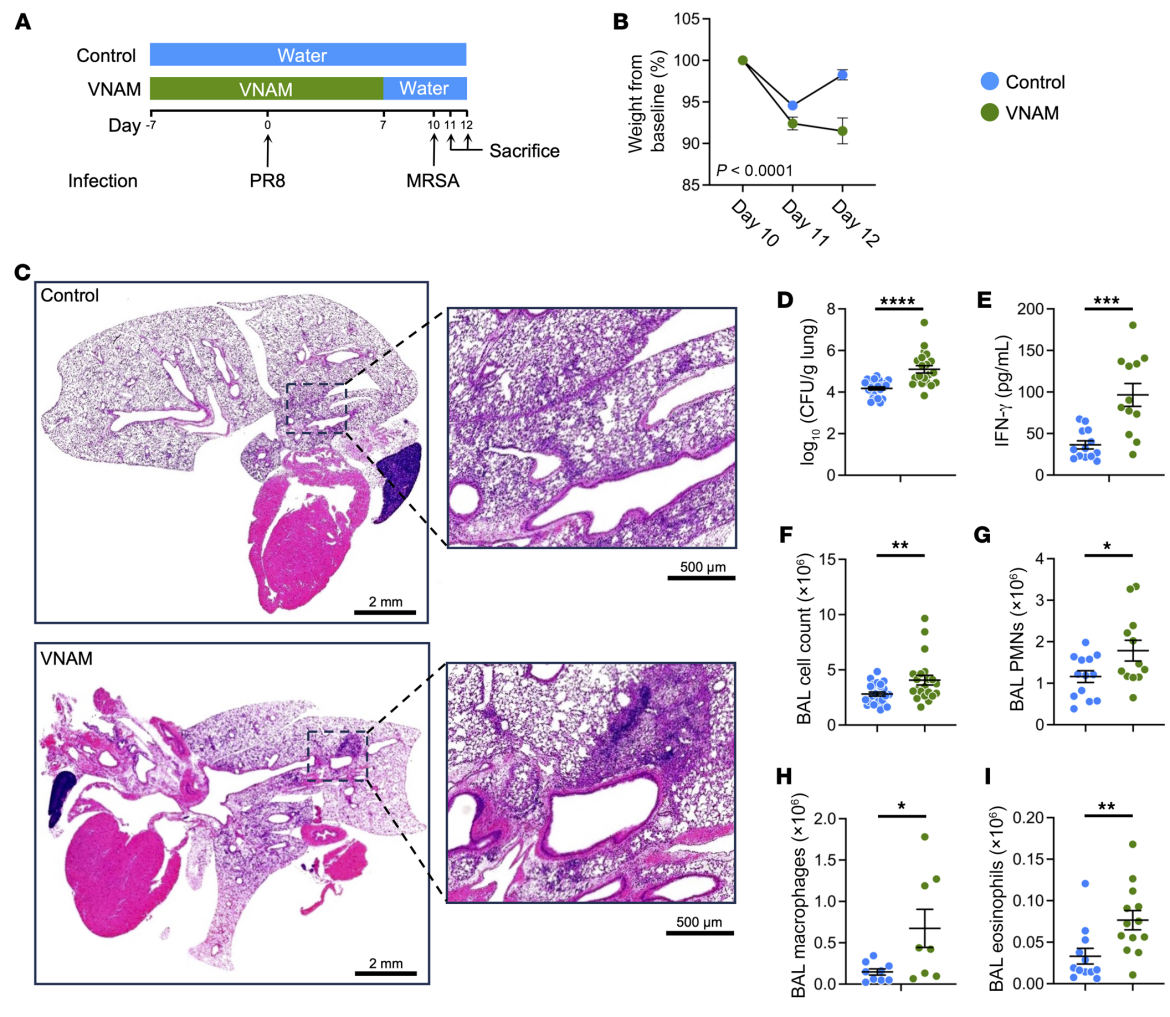
Antibiotic treatment of mice during influenza infection impairs bacterial clearance and augments lung inflammation after a subsequent challenge with MRSA. (**A**) Mice were infected with influenza (PR8, 500 PFU) at day 0 followed by MRSA at day 10. Control or antibiotics (VNAM) were started 7 days before PR8 infection to allow mice to equilibrate to the treatment and discontinued at day 7 to allow it to wash out before MRSA challenge. (**B**) Weight relative to that at day 10 showed a slower recovery after MRSA challenge in the VNAM-treated group (*n* = 18) compared with control (*n* = 31) at days 11 and 12 (1 and 2 days after MRSA infection, respectively). (**C**) Representative images from 3 different H&E-stained lungs at day 11 of the 2-hit model. Scale bars: 2 mm (left), 500 μm (right). (**D**–**I**) Mice were sacrificed on day 12 after the 2-hit infection; the lungs were evaluated for CFU of bacteria (*n* = 26 and 21 for control and VNAM groups, respectively) (**D**), and BAL was evaluated for IFN-γ levels (*n* = 13 and 12 for control and VNAM groups, respectively) (**E**), total cell count (*n* = 26 and 21 for control and VNAM groups, respectively) (**F**), PMN count (*n* = 13 and 12 for control and VNAM groups, respectively) (**G**), macrophages (*n* = 9 and 8 for control and VNAM groups, respectively) (**H**), and eosinophils (*n* = 12 and 13 for control and VNAM groups, respectively) (**I**). *P < 0.05, **P < 0.01, ***P < 0.005, ****P < 0.0001 by 2-tailed Student’s *t* test.

The onset of a secondary bacterial infection after the initial viral illness is classically described as the recrudescence of symptoms (i.e., redevelopment of respiratory illness after an initial phase of recovery) (15). To replicate this clinical scenario, we established a murine model where mice were infected with influenza (PR8), which peaks in severity on day 7 after infection, followed by a subsequent MRSA challenge at day 10 (Figure 1A) (16). MRSA was used because recent epidemiological evidence indicates that *S.* *aureus* is the most common pathogen causing a secondary bacterial pneumonia (4, 17). Additionally, mice were treated with either control or broad-spectrum antibiotics in the drinking water starting at 7 days before PR8 infection to allow animals to acclimate to VNAM (vancomycin, neomycin, ampicillin, and metronidazole).

With the 2-hit model, VNAM-treated compared with control mice had significantly greater weight loss (Figure 1B) and more histological evidence of lung injury (Figure 1C). Additionally, antibiotic treatment during influenza infection had a paradoxical effect on a subsequent bacterial challenge with the VNAM group having higher MRSA colony-forming units (CFU) in the lungs compared with control (Figure 1D). IFN-γ was also elevated in the bronchoalveolar lavage (BAL) of VNAM versus control 2 days after MRSA infection (Figure 1E). Furthermore, VNAM-treated mice had higher total cell count, neutrophils, macrophages, and eosinophils in the BAL compared with control (Figure 1, F–I, and Supplemental Figure 1; supplemental material available online with this article; https://doi.org/10.1172/JCI180986DS1). These data suggest that antibiotic treatment during influenza infection impairs MRSA clearance.

Notably, the increased lung injury caused by antibiotic use was specific to the 2-hit model. VNAM had no effect on bacterial clearance and lung inflammation from MRSA infection alone (Supplemental Figure 2). Similarly, influenza alone had similar levels of lung inflammation between both control and VNAM conditions (Supplemental Figure 3). We also performed the 2-hit model using PR8 infection followed by *S.* *pneumoniae* and found that VNAM treatment increased lung inflammation compared with control (Supplemental Figure 4), suggesting that the detrimental effects of antibiotics are generalized to the various causes of bacterial superinfections. Furthermore, no difference in lung inflammation was noted on day 10 after influenza infection (Supplemental Figure 5), which is the time point of MRSA infection in the 2-hit model, further supporting that the effect of antibiotics is specific to post-influenza MRSA pneumonia. Altogether, these data indicate that antibiotic use during influenza infection has negative consequences that diminish bacterial clearance and worsen lung injury after a secondary bacterial challenge.

### Antibiotic use during influenza infection augments lung eosinophils, which reduce MRSA clearance and increase lung injury.

**Figure 2:**
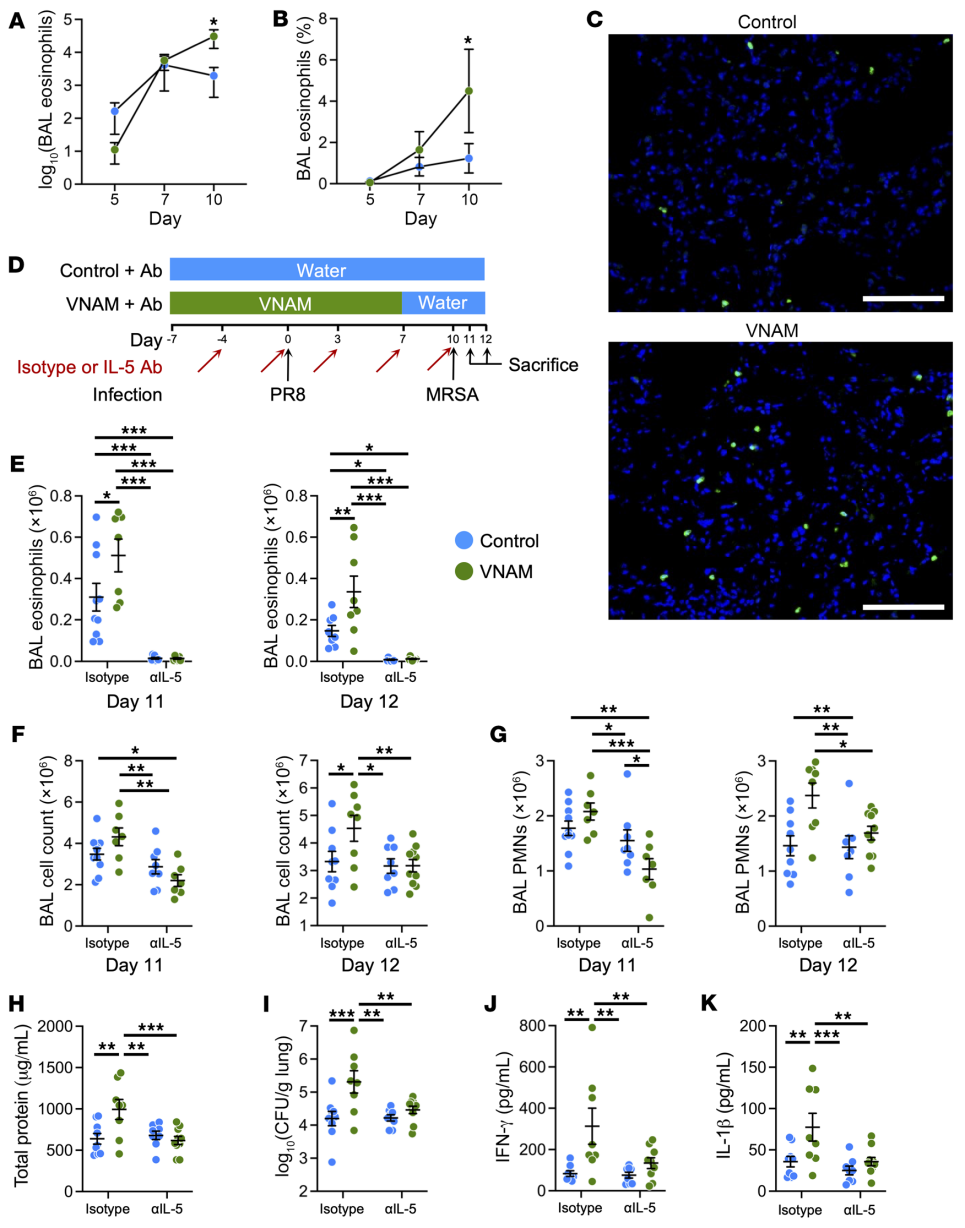
Depletion of eosinophils improves bacterial clearance and lung inflammation in the antibiotic-treated group injured with influenza followed by MRSA. (**A** and **B**) BAL eosinophil numbers (**A**) and percentages (**B**) in control and VNAM-treated mice infected with influenza (PR8, 250 PFU) (*n* = 3–4). (**C**) EoCre × ROSA^mTmG^ transgenic mice were treated with control or VNAM and then injured with influenza (PR8, 250 PFU). Mice were sacrificed on day 10 after influenza infection, and lung slices were immunostained for GFP (eosinophils; green) and DAPI (blue). Image is representative of 3 different samples. Scale bars: 100 μm. (**D**) Mice were infected with influenza (PR8, 250 PFU) at day 0 followed by MRSA at day 10. Control or antibiotics (VNAM) were started 7 days before PR8 infection to allow mice to equilibrate to the treatment and discontinued at day 7 to allow it to wash out before MRSA challenge. Mice were given intraperitoneal injections of either an isotype antibody (*n* = 7–10) or an anti–IL-5 antibody (*n* = 7–10) every 3–4 days. (**E**) BAL eosinophils were effectively depleted after IL-5 antibody treatment. (**F** and **G**) Total cell count (**F**) and PMNs (**G**) in the BAL were evaluated 1 and 2 days after MRSA infection (days 11 and 12, respectively). (**H**–**K**) Mice were sacrificed on day 12 and evaluated for total protein in the BAL (**H**), CFU of bacteria in the lungs (**I**), BAL IFN-γ levels (**J**), and BAL IL-1β levels (**K**). *P < 0.05, **P < 0.01, ***P < 0.001 by 2-way ANOVA.

Lung eosinophils increased in the lungs over the time course of the influenza infection but were higher in VNAM-treated mice compared with control (Figure 1I and Figure 2, A–C). Therefore, we tested whether eosinophils were the effectors that impaired lung innate immunity caused by antibiotics during the PR8-MRSA injury. Mice were treated with either an isotype or an anti–IL-5 antibody (Figure 2D), which effectively suppressed lung eosinophils (Figure 2E). Eosinophil depletion attenuated the accumulation of inflammatory cells in the BAL (Figure 2, F and G) and improved lung barrier function (Figure 2H). IL-5 inhibition also reduced the MRSA burden in the lungs (Figure 2I) and BAL levels of IFN-γ and IL-1β (Figure 2, J and K). These data support the concept that antibiotic-induced eosinophils mediate the reduced ability to clear MRSA and worsened lung inflammation after influenza infection.

### Antibiotic treatment during influenza infection impairs macrophage function in vivo.

**Figure 3:**
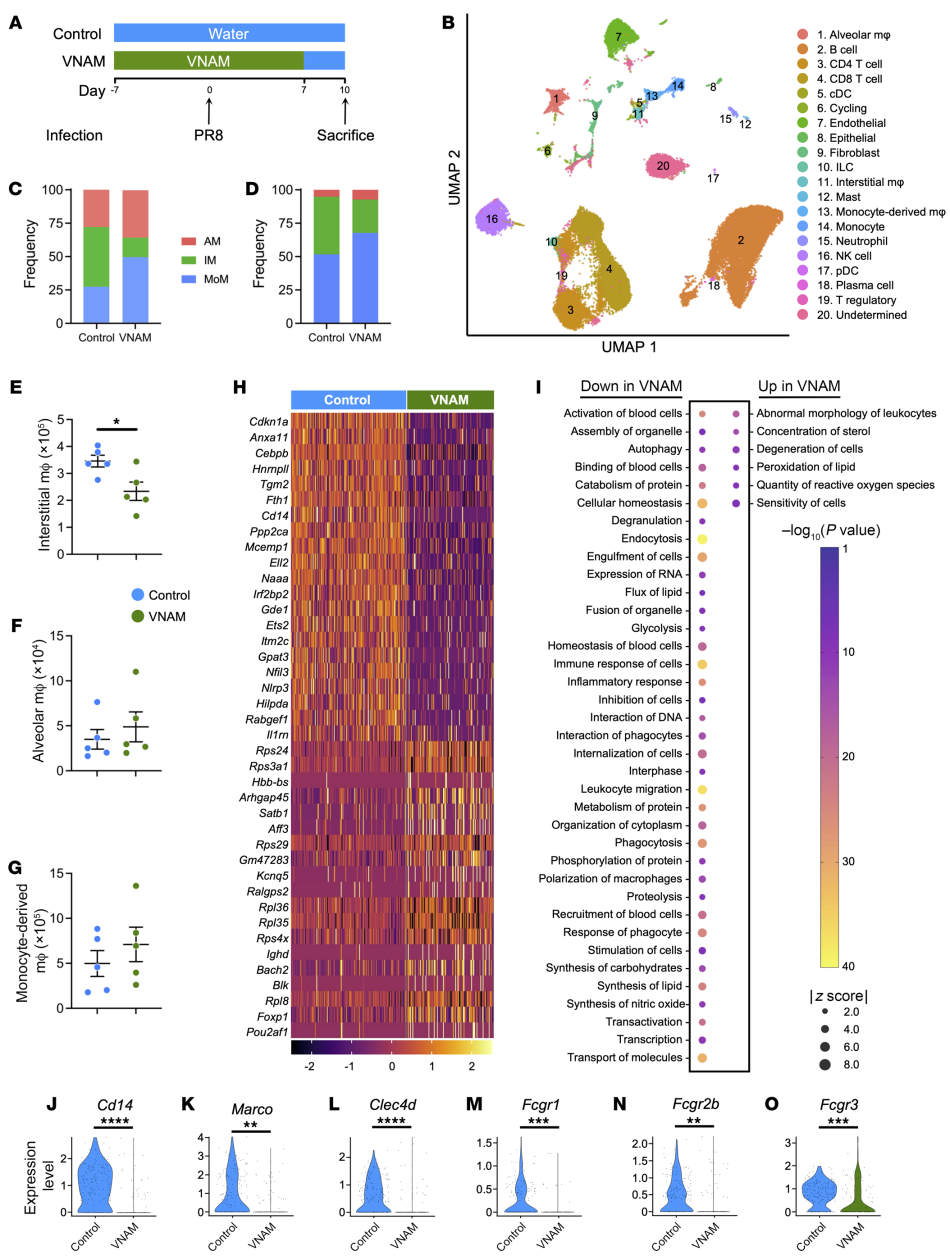
Antibiotic treatment of mice during influenza infection attenuates interstitial macrophage numbers and causes transcriptomic changes in alveolar macrophages consistent with an immunosuppressive phenotype. (**A**) Mice were infected with influenza (PR8, 250 PFU) at day 0. Control or antibiotics (VNAM) were started 7 days before PR8 infection to allow mice to equilibrate to the treatment and discontinued at day 7. (**B**–**O**) Lungs from mice sacrificed at day 10 were processed for scRNA-Seq (**B**, **C**, and **H**–**O**) and flow cytometry (**D**–**G**). (**B**) UMAP visualization of the major cell populations identified in the transcriptomic data set. (**C** and **D**) Relative numbers of alveolar macrophages (AM), interstitial macrophages (IM), and monocyte-derived macrophages (MoM) between control and VNAM groups in the scRNA-Seq data (**C**) and by flow cytometry analysis (**D**). (**E**–**G**) Flow cytometry analysis (*n* = 5) for total number of interstitial macrophages (*P < 0.05 by 2-tailed Student’s *t* test) (**E**), alveolar macrophages (**F**), and monocyte-derived macrophages (**G**). (**H**) Heatmap of the top 20 (by lowest FDR) differentially expressed genes (DEGs) in alveolar macrophages between control and VNAM-treated mice. The entire DEG list is provided in Supplemental Table 1. (**I**) DEGs in tissue-resident alveolar macrophages between control and VNAM groups were evaluated with Ingenuity Pathway Analysis (IPA). Disease and Function analysis was performed, and only nonredundant, downstream functional pathways (|*z* score| > 2) were visualized. The complete Disease and Function analysis is provided in Supplemental Table 2. (**J**–**O**) Tissue-resident alveolar macrophage expression of the phagocytosis receptors in control (*n* = 319) and VNAM (*n* = 316) conditions: *Cd14* (**J**), *Marco* (**K**), *Clec4d* (**L**), *Fcgr1* (**M**), *Fcgr2b* (**N**), and *Fcgr3* (**O**). The complete list of DEGs between control and VNAM in alveolar macrophages is given in Supplemental Table 1. Adjusted *P* value: *P < 0.05, **P < 0.01, ***P < 0.001, ****P < 0.0001. mϕ, macrophage.

The alveolar macrophage plays a predominant role in bacterial phagocytosis and clearance within the airspaces (18, 19). To evaluate whether antibiotics affect the alveolar macrophage transcriptome in vivo, we performed single-cell RNA sequencing (scRNA-Seq) of the lungs from control and VNAM conditions (Figure 3). The VNAM effects on lung immunity during influenza infection were seen within 1 day after MRSA infection (Figure 1), suggesting that changes were present by the time of the bacterial challenge. Thus, we chose day 10 after influenza infection (without an MRSA challenge) for the scRNA-Seq analysis (Figure 3A).

Although we were able to identify most immune cell types in the lungs after influenza infection (Figure 3B and Supplemental Figure 6), eosinophils were not found, owing to their fragility and high RNase content that impede identification by scRNA-Seq analysis (20, 21). Therefore, we focus on macrophages, which are important phagocytes that mediate pathogen clearance in the lungs (22). We identified tissue-resident alveolar macrophages, interstitial macrophages, and monocyte-derived macrophages in the scRNA-Seq data set, and a relative decrease in interstitial macrophage population was noted in the VNAM-treated group compared with control (Figure 3C). Flow cytometry analysis of lung macrophage subsets after influenza infection confirmed lower interstitial macrophage numbers after VNAM treatment compared with control (Figure 3, D–G, and Supplemental Figure 7).

Next, differentially expressed genes (DEGs) in macrophage subsets between the control and VNAM conditions were determined (Supplemental Table 1). We found 673 genes in tissue-resident alveolar macrophages to be significantly different (adjusted *P* < 0.01) between control and VNAM groups (Figure 3H and Supplemental Table 1). However, only 29 and 4 DEGs were identified in interstitial macrophages and monocyte-derived macrophages, respectively (Supplemental Table 1). Because the transcriptome was largely unchanged in interstitial and monocyte-derived macrophages between control and VNAM conditions, we focused our downstream analysis on the DEGs in alveolar macrophages.

Ingenuity Pathway Analysis (IPA) was performed using the DEGs between control and VNAM in alveolar macrophages to understand the signaling implications of the transcriptomic changes (Supplemental Table 2). We used the Disease and Function Analysis module in IPA to determine the predicted downstream functional alterations caused by antibiotic treatment (Figure 3I). The analysis demonstrated the negative immunomodulatory effects of antibiotic treatment during influenza infection with suppression of pertinent immunologic functions in the VNAM group, such as “engulfment of cells,” “immune response of cells,” “leukocyte migration,” “phagocytosis,” “recruitment of blood cells,” and “response of phagocytes.” Accordingly, we evaluated expression of various phagocytosis receptors and found that alveolar macrophages in VNAM-treated mice had lower expression of *Cd14*, *Marco*, *Clec4d*, *Fcgr1*, *Fcgr2b*, and *Fcgr3* (Figure 3, J–O).

These data support the concept that antibiotic use during influenza infection impairs lung immunity potentially through reduction of interstitial macrophage numbers and impairment of alveolar macrophage phagocytosis, which could result in an attenuated ability to clear potential pathogens from the airspaces.

### Antibiotic-induced lung eosinophils impair macrophage clearance of bacteria through the secretion of MBP-1.

**Figure 4:**
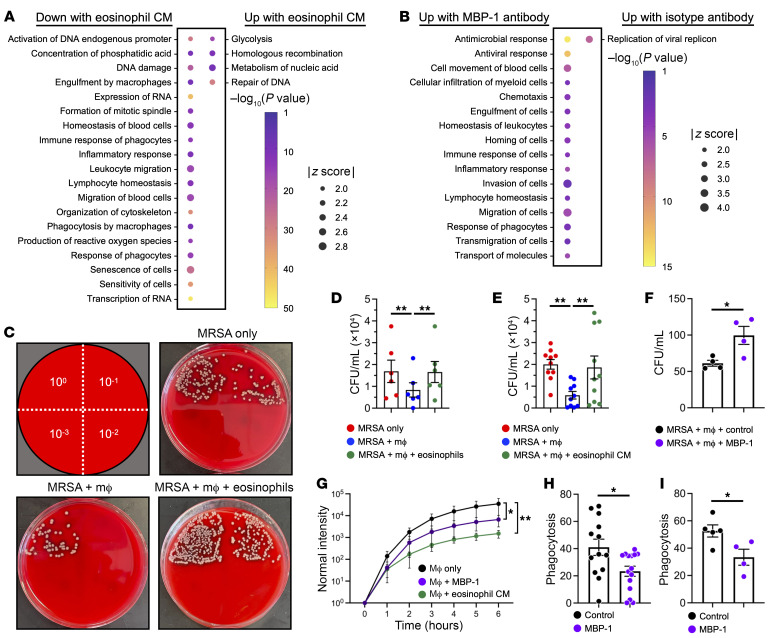
Eosinophils suppress macrophage phagocytosis through secretion of MBP-1. (**A** and **B**) Macrophages (RAW 264.7 cells) were processed for RNA-Seq. DEGs (see Supplemental Table 3 for entire DEG list) were analyzed with IPA. (**A**) Macrophages were cultured in conditioned medium from either eosinophils or epithelial cells as a control. Disease and Function Analysis was performed, and nonredundant pathways (|*z* score| > 2) were visualized. See Supplemental Table 4 for the complete list of pathways. (**B**) Macrophages were cultured in eosinophil conditioned medium with addition of an isotype or anti–MBP-1 antibody. Disease and Function Analysis was performed, and nonredundant pathways (|*z* score| > 2) were visualized. See Supplemental Table 4 for the complete list. (**C**) MRSA was cultured alone, with macrophages, or with macrophages and primary eosinophils for 4 hours and MRSA CFU was calculated. (**D**) Quantification of MRSA CFU (*n* = 6). **P < 0.01 by repeated-measures 1-way ANOVA and post hoc analysis. (**E**) CFU of MRSA cultured for 4 hours alone, with macrophages, or with macrophages and conditioned medium from primary eosinophils (*n* = 11). **P < 0.01 by repeated-measures 1-way ANOVA and post hoc analysis. (**F**) CFU of MRSA cultured for 2 hours with macrophages plus addition of control (BSA) or recombinant MBP-1 (*n* = 4). *P < 0.05 by 2-tailed Student’s *t* test. (**G**) Fluorescence intensity over time reflects phagocytosis of pHrodo-labeled *S.* *aureus* bioparticles (*n* = 4). *P < 0.05, **P < 0.01 by repeated-measures 2-way ANOVA. (**H** and **I**) Effect of MBP-1 on phagocytosis of *S.* *aureus* bioparticles by murine alveolar macrophages in vivo (control, *n* = 13; MBP-1, *n* = 15) (**H**) and in an ex vivo PCLS culture (control, *n* = 5; MBP-1, *n* = 4) (**I**). *P < 0.05 by 2-tailed Student’s *t* test.

Given our observations that antibiotic treatment during influenza infection augments lung eosinophils (Figure 2) and the transcriptomic data suggesting impairment of alveolar macrophage phagocytosis (Figure 3), we postulated that eosinophils negatively regulate the behavior of macrophages and suppress their ability to clear bacteria during influenza infection. To test this hypothesis, we cultured macrophages in conditioned medium from eosinophils versus control and evaluated transcriptomic changes. We used the Disease and Function Analysis in IPA to evaluate the DEGs (Supplemental Table 3) that were significantly different between conditions and found that the addition of eosinophil conditioned medium suppressed immunologic pathways in the macrophages including “engulfment by macrophages,” “immune response of phagocytes,” “phagocytosis by macrophages,” and “response of phagocytes” (Figure 4A and Supplemental Table 4). Similar to the findings in alveolar macrophages (Figure 3), eosinophil conditioned medium attenuated expression of several phagocytosis receptors (Supplemental Figure 8).

The ability of eosinophil conditioned medium to negatively affect macrophage function suggested that eosinophils facilitate the effect through secreted factors. Eosinophils release prepackaged cationic proteins such as MBP-1 to mediate their immunomodulatory effects (23). As such, we tested whether MBP-1 secretion could suppress macrophage functions by adding an anti–MBP-1 antibody to the eosinophil conditioned medium. Compared with an isotype antibody, MBP-1 blockade reversed the inhibitory effect of eosinophil conditioned medium on macrophage function. Indeed, pathways such as “antimicrobial response,” “antiviral response,” “cell movement of blood cells,” “cell infiltration of myeloid cells,” “engulfment of cells,” “immune response of cells,” “migration of cells,” and “response of phagocytes” were augmented by MBP-1 inhibition in eosinophil conditioned medium (Figure 4B and Supplemental Table 4). Similarly, MBP-1 blockade reversed the effects of eosinophil conditioned medium on expression of *Fcgr1*, *Fcgr2b*, and *Fcgr3* (Supplemental Figure 8).

The transcriptomic analyses suggest that eosinophils suppress immunologic pathways in macrophages needed for bacterial clearance. Therefore, we established a bacterial killing assay in which macrophages were cultured with MRSA either alone or in the presence of eosinophils (Figure 4C). Whereas macrophages suppressed bacterial growth, both eosinophils and eosinophil conditioned medium antagonized macrophages such that bacterial CFUs were similar to MRSA-only conditions (Figure 4, D and E, respectively). To determine how eosinophils impaired macrophage ability to suppress MRSA growth, we added recombinant MBP-1 to bacteria and macrophage cocultures, which augmented MRSA counts compared with control conditions (Figure 4F). Pretreatment of macrophages or MRSA with recombinant MBP-1 with removal during coculture had no effect on macrophage suppression of bacteria growth (Supplemental Figure 9, A and B). Moreover, MBP-1 treatment of MRSA alone had no effect on bacterial growth (Supplemental Figure 9C). These data are congruous with the transcriptomic evaluation and demonstrate eosinophil suppression of macrophage function via MBP-1 secretion.

Notably, alveolar macrophages in VNAM-treated mice compared with control had suppression of phagocytosis pathways (Figure 3D) and receptors (Figure 3, E–J). Similarly, macrophages treated with eosinophil conditioned medium had suppressed phagocytosis by transcriptomic evaluation (Figure 4A and Supplemental Figure 8). Therefore, we evaluated macrophage engulfment of *S.* *aureus* bioparticles and found that the addition of recombinant MBP-1 or eosinophil conditioned medium impaired phagocytosis (Figure 4G). Because the scRNA-Seq suggested that VNAM treatment has specific effects on tissue-resident alveolar macrophages, we developed phagocytosis assays that used primary tissue-resident alveolar macrophages. Alveolar macrophages change their phenotype when removed from their native microenvironment (24–27). Therefore, we developed assays that maintained these cells in the lung niche. First, we instilled *S.* *aureus* bioparticles into the lungs of mice and found that coadministration of recombinant MBP-1 suppressed phagocytosis by alveolar macrophages in vivo (Figure 4H). Next, we created a phagocytosis assay using precision-cut lung slice cultures of mouse lungs, which contain alveolar macrophages. Consistent with our previously described results, MBP-1 suppressed phagocytosis of *S.* *aureus* bioparticles compared with control (Figure 4I). These data indicate that eosinophils inhibit alveolar macrophage ability to phagocytose and suppress bacterial growth through the release of MBP-1.

### Antibiotics cause a fungal dysbiosis that augments lung eosinophilia and lung injury.

**Figure 5:**
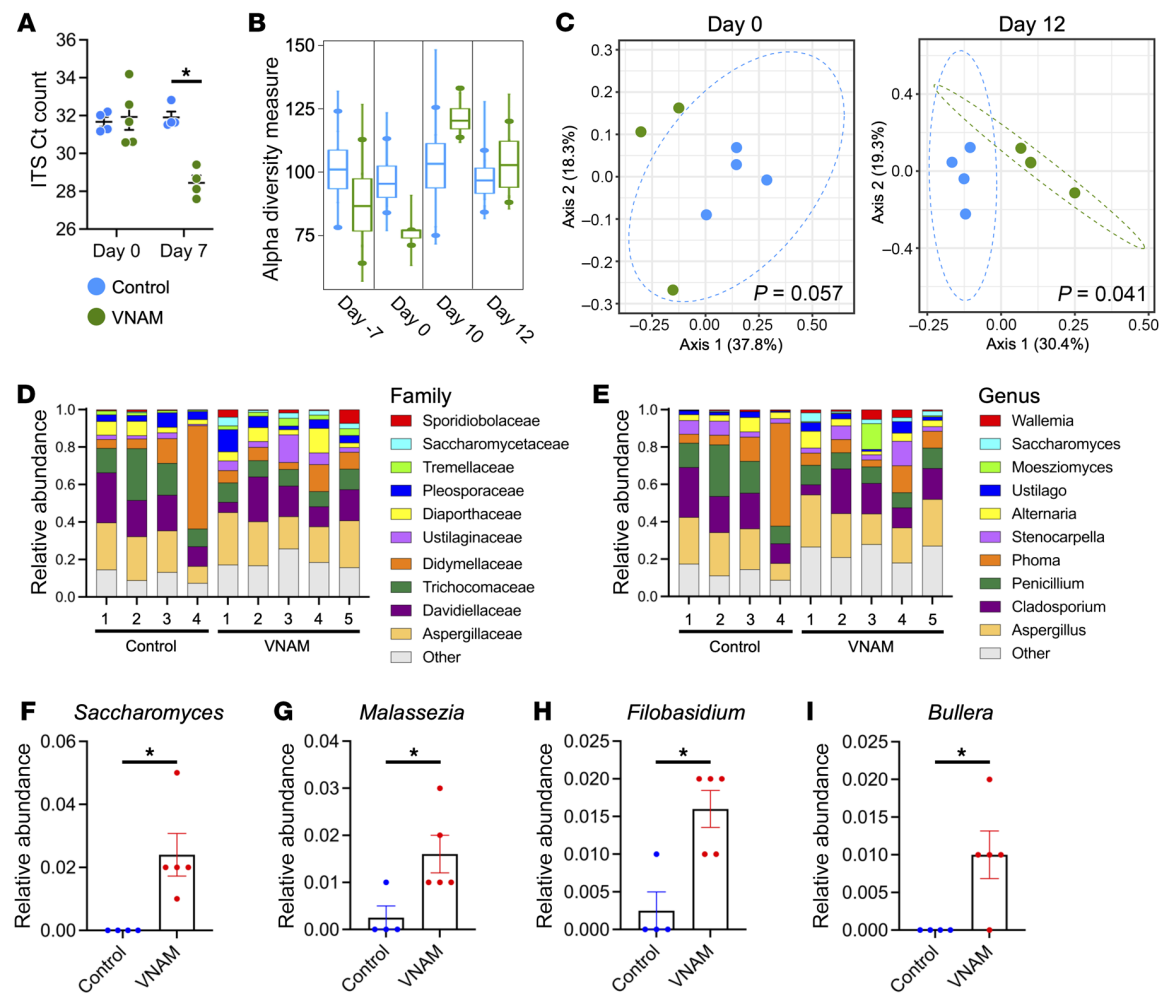
Antibiotics cause fungal dysbiosis during the influenza-MRSA 2-hit challenge. (**A**) Stool was collected at days 0 and 7 from VNAM (*n* = 5 and 4, respectively) and control (*n* = 4) groups for ITS PCR. *P < 0.001 by 2-tailed Student’s *t* test. (**B**–**I**) ITS sequencing of the stool collected at the designated time points from control and VNAM-treated mice infected with influenza (day 0) followed by MRSA (day 10). (**B**) Chao index showed no difference in α-diversity (*n* = 3–4). (**C**) Principal coordinates analysis demonstrated changes in β-diversity at day 12 (*n* = 3–4). (**D** and **E**) Relative abundance for individual samples at day 10 of the 2-hit model in control (*n* = 4) and VNAM (*n* = 5) groups at the family (**D**) and genus (**E**) levels. The top 10 families or genera are shown, and all others are cumulatively reported in the “Other” group. (**F**–**I**) The relative abundance at the genus level was significantly higher in the VNAM (*n* = 5) compared with the control (*n* = 4) group for *Saccharomyces* (**F**), *Malassezia* (**G**), *Filobasidium* (**H**), and *Bullera* (**I**). *P < 0.05 by 2-tailed Student’s *t* test.

Next, we wanted to understand the mechanism by which antibiotics caused eosinophils to accumulate in the lungs during influenza infection. Antibiotics perturb the homeostatic interactions between the microbiome and host (28), so we postulated that gut dysbiosis contributed to lung eosinophilia. As expected, broad antibacterial effects of VNAM depleted the gut of bacterial microbiota and induced gut dysbiosis (Supplemental Figure 10 and Supplemental Table 5). Disrupting one niche of the microbiome ecosystem can impact another (29, 30). In particular, antibiotic suppression of the bacterial microbiome provides a permissive environment that allows fungi (i.e., the mycobiome) to expand (31). Indeed, VNAM treatment caused a more than 11-fold increase in fungal burden compared with control (Figure 5A).

To characterize the gut fungal dysbiosis, we performed internal transcribed spacer (ITS) sequencing of the stool from control and VNAM-treated mice infected with influenza (Supplemental Table 5). The α-diversity of fungal species had little change between conditions at all time points (Figure 5B). In contrast, the mycobiome β-diversity was significantly different between control and VNAM (Figure 5C). The ITS sequencing revealed changes in the relative abundance of intestinal fungi (Figure 5, D and E). However, significant differences between control and VNAM groups were found with a few genera, such as *Saccharomyces* (Figure 5F), *Malassezia* (Figure 5G), *Filobasidium* (Figure 5H), and *Bullera* (Figure 5I).

**Figure 6:**
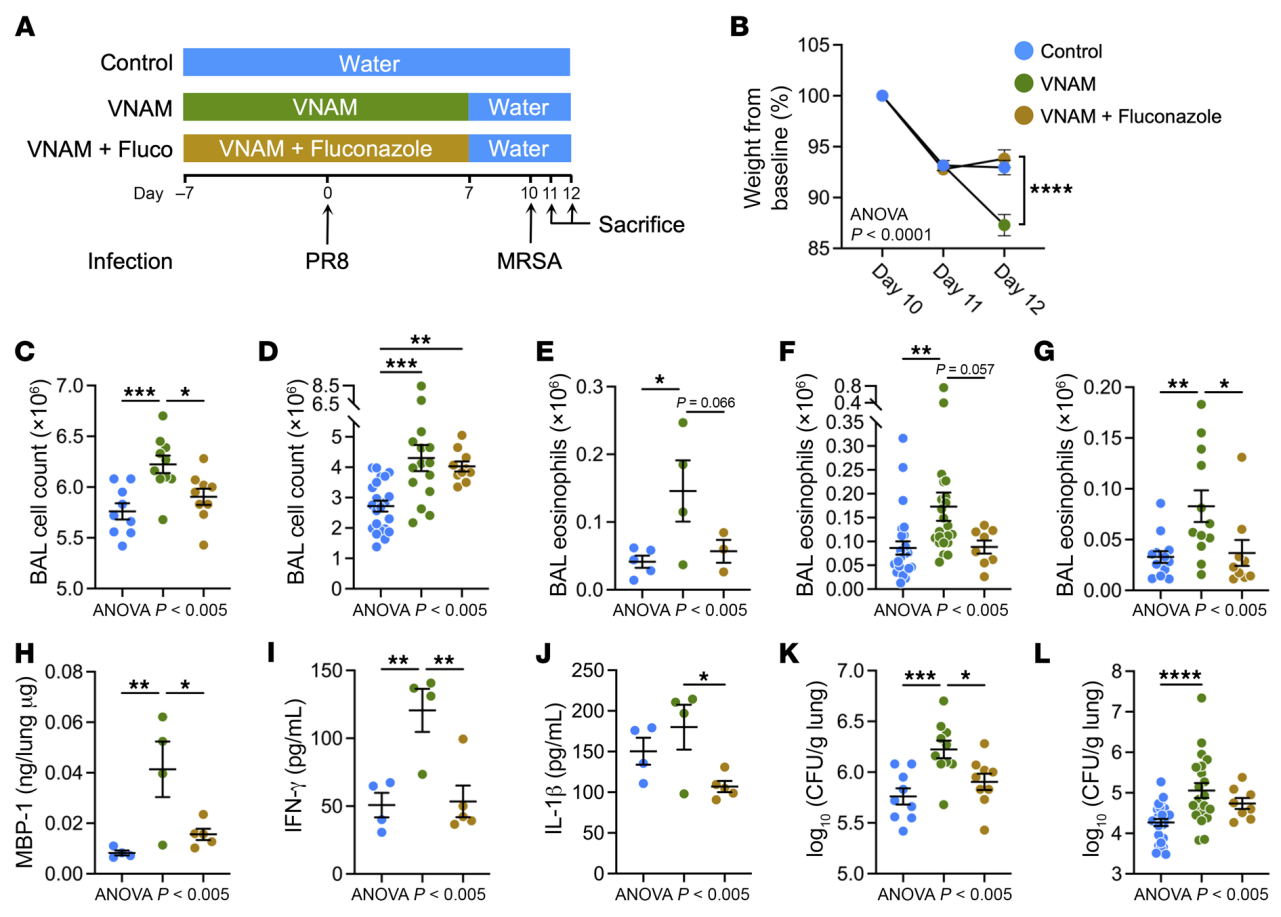
Cotreatment with fluconazole improves bacterial clearance and reverses the worsened lung injury in antibiotic-treated mice. (**A**) Mice were infected with influenza (PR8, 250 PFU) at day 0 followed by MRSA at day 10. Control, antibiotics alone (VNAM), or VNAM and cotreatment with fluconazole (VNAM+Fluco) were started 7 days before PR8 infection to allow mice to equilibrate to the treatment and discontinued at day 7 to allow it to wash out before MRSA challenge. (**B**) Weight relative to that on day 10 showed slower recovery after MRSA challenge in the VNAM-treated group (*n* = 22) compared with control (*n* = 21) and VNAM+Fluco (*n* = 13) groups at days 11 and 12 (1 and 2 days after MRSA infection, respectively) by 2-way ANOVA. ****P < 0.0001 in post hoc analysis at day 12. (**C**–**L**) Mice in control (C), VNAM (V), and VNAM+Fluco (VF) groups were injured in the 2-hit model and sacrificed for evaluation of (**C**) day 11 BAL total cell count (C: *n* = 9; V: *n* = 10; VF: *n* = 10); (**D**) day 12 BAL cell count (C: *n* = 22; V: *n* = 15; VF: *n* = 10); (**E**) day 10 BAL eosinophil count (C: *n* = 5; V: *n* = 4; VF: *n* = 3); (**F**) day 11 BAL eosinophil count (C: *n* = 26; V: *n* = 24; VF: *n* = 8); (**G**) day 12 BAL eosinophil count (C: *n* = 13; V: *n* = 12; VF: *n* = 9); (**H**) day 10 lung MBP-1 levels (C: *n* = 4; V: *n* = 4; VF: *n* = 5); (**I**) day 12 BAL IFN-γ levels (C: *n* = 4; V: *n* = 4; VF: *n* = 5); (**J**) day 12 BAL IL-1β levels (C: *n* = 4; V: *n* = 4; VF: *n* = 5); (**K**) day 11 lung MRSA CFU (C: *n* = 9; V: *n* = 10; VF: *n* = 9); and (**L**) day 12 lung MRSA CFU (C: *n* = 27; V: *n* = 21; VF: *n* = 8). (**C**–**L**) One-way ANOVA with post hoc analysis was used to determine *P* values within the respective panels. ANOVA *P* values are listed in each panel. Post hoc comparisons are represented as follows: *P < 0.05, **P < 0.01, ***P < 0.001, ****P < 0.0001.

We and others have demonstrated that gut fungal dysbiosis can have distal effects in the lungs and exacerbate allergic airways disease (32–35). Therefore, we postulated that antibiotic-induced expansion of the gut mycobiome could also affect the lung innate immunity required for clearance of bacteria that are aspirated into the lower airspaces. The VNAM group was concomitantly treated with fluconazole, an antifungal agent, to suppress fungal overgrowth during antibiotic administration (Figure 6A). We found that the group with VNAM and fluconazole cotreatment was similar to the controls and had significantly less weight loss and fewer inflammatory cells in the BAL compared with the VNAM group (Figure 6, B–D). Notably, fluconazole treatment reversed the lung eosinophilia caused by VNAM during influenza infection (Figure 6, E–G). Moreover, we also found that MBP-1 levels, which increased with VNAM treatment compared with control, were reduced with cotreatment with fluconazole (Figure 6H). The VNAM plus fluconazole group also had reduced IFN-γ (Figure 6I), IL-1β (Figure 6J), and MRSA CFU in the lungs (Figure 6, K and L) when compared with VNAM-only treatment. These data demonstrated that antibiotics augment lung eosinophils and lung inflammation whereas suppression of fungal expansion with fluconazole attenuated the effect.

Two microbiome compartments, the lung and the gut, have important roles in lung health and disease (28). Accordingly, we repeated the fungal suppression experiments by cotreating with VNAM and oral amphotericin, an antifungal that is poorly absorbed by the gut and, hence, will attenuate gut fungi and have minimal effects in the lungs. Amphotericin treatment led to significant improvement compared with VNAM alone, which phenocopied the fluconazole experiments (Supplemental Figure 11). Altogether, these data suggest that antibiotic use during influenza infection causes expansion of the gut mycobiome driven by multiple fungal organisms. The fungal expansion has distal effects in the lungs (i.e., gut-lung axis) and augments the number of lung eosinophils, which our prior results (Figure 4) indicate can suppress macrophage phagocytosis of MRSA.

### Eosinophil levels correlate with increased length of stay and more inflammation in hospitalized patients.

**Figure 7:**
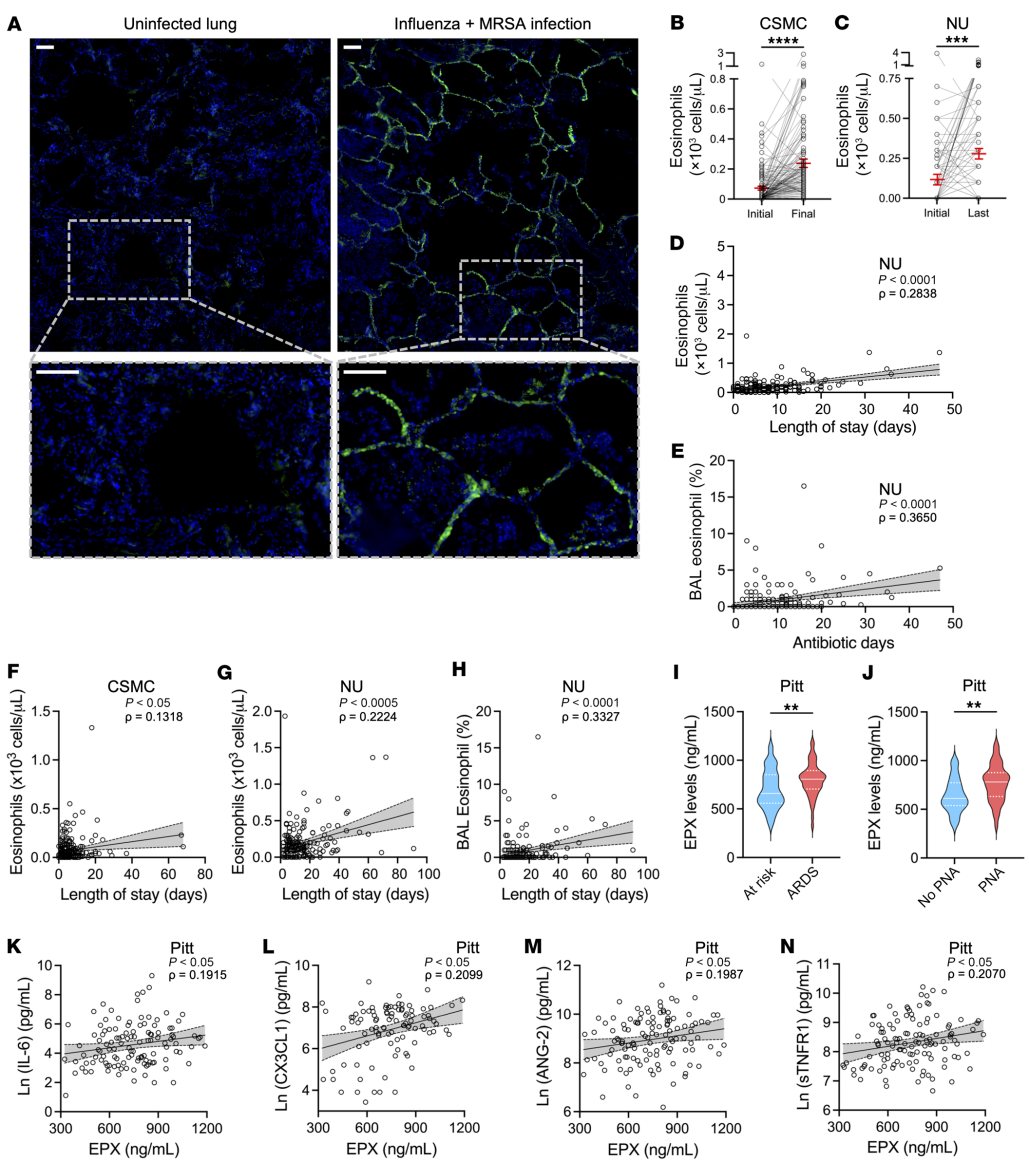
Eosinophils correlate with antibiotic use and worsen clinical outcomes in hospitalized patients. (**A**) Lungs from an uninfected patient and one who died from influenza followed by MRSA infection were immunostained for MBP-1 to identify eosinophils (green fluorescence) and counterstained with DAPI (blue). See Supplemental Figure 12 for a representative image of the lungs from a patient who died from influenza with *S.* *pneumoniae* superinfection. Scale bars: 100 μm. (**B**) Initial and final eosinophil levels in peripheral blood of patients hospitalized for influenza infection on antibiotics for more than 4 days (*n* = 131). (**C**) Initial and final eosinophil levels in peripheral blood of ICU patients requiring mechanical ventilation and on antibiotics for more than 4 days (*n* = 121). (**D** and **E**) Mean eosinophils in peripheral blood (*n* = 175) (**D**) and BAL (**E**) correlated with days of antibiotic use in the ICU (*n* = 186). (**F**) Mean eosinophils in peripheral blood correlated with hospital length of stay for influenza patients (*n* = 229). (**G** and **H**) Mean eosinophils in peripheral blood (*n* = 175) (**G**) and BAL (*n* = 186) (**H**) correlated with length of stay in the ICU. (**I**–**N**) Eosinophil peroxidase (EPX) was measured in plasma of ICU patients. (**I** and **J**) Violin plot of EPX levels in ICU patients with ARDS (*n* = 51) compared with those at risk (*n* = 67) (**I**) and with pneumonia (PNA; *n* = 96) compared with those without pneumonia (No PNA; *n* = 23) (**J**). (**K**–**N**) EPX levels were correlated with plasma levels (*n* = 119) of IL-6 (**K**), CX3CL1 (**L**), ANG-2 (**M**), and soluble TNFR1 (**N**). Data presented from Cedars-Sinai Medical Center (CSMC) (**B** and **F**), Northwestern University (NU) (**C**–**E**, **G**, and **H**), and University of Pittsburgh (Pitt) (**I**–**N**). The data presented include all the patients within respective cohorts. Patient demographics for each cohort are described in Supplemental Table 7. **P < 0.01, ***P < 0.001, ****P < 0.0001 by 2-tailed Student’s *t* test (**B**, **C**, **I**, and **J**). Spearman’s correlation was used to determine association between various parameters (**D**–**H** and **K**–**N**).

Our data suggest that eosinophils may have negative effects on lung immunity. We immunostained for MBP-1 in the lungs of a patient who died from a secondary bacterial pneumonia after influenza infection and identified many eosinophils (Figure 7A and Supplemental Figure 12). Next, we evaluated data from patients admitted with an influenza infection at Cedars-Sinai Medical Center and found an increase in peripheral eosinophil counts in those treated with antibiotics (Figure 7B and Supplemental Table 6). Furthermore, we evaluated a cohort of patients hospitalized in the Northwestern Memorial Hospital intensive care unit (ICU) who required mechanical ventilation and found a similar increase in eosinophil counts with antibiotic administration (Figure 7C and Supplemental Table 6). Additionally, days of antibiotic use during the ICU hospitalization positively correlated with peripheral eosinophil counts (Figure 7D) and BAL eosinophil percentage (Figure 7E).

Because our data demonstrated that eosinophils impair alveolar macrophages from clearing MRSA, we predicted that elevated eosinophil levels may be a negative prognostic factor for patients hospitalized for influenza. Indeed, peripheral blood eosinophil levels were associated with a longer length of stay (LOS) in the hospital for influenza (Figure 7F). Furthermore, eosinophil levels in the blood (Figure 7G) and lungs (Figure 7H) positively correlated with the ICU LOS. Altogether, these findings demonstrate that eosinophils significantly increase with antibiotic use and are associated with increased LOS.

Next, in a cohort of mechanically ventilated patients with acute respiratory failure at the University of Pittsburgh Medical Center (Supplemental Table 6), we measured plasma eosinophil peroxidase (EPX) as a surrogate marker for eosinophil levels (36). Plasma EPX levels were higher in patients with acute respiratory distress syndrome (ARDS) versus those at risk (Figure 7I). Patients with a diagnosis of pneumonia also had higher EPX plasma levels compared with those without pneumonia (Figure 7J). Furthermore, plasma EPX levels positively correlated with several inflammation and injury biomarkers, such as IL-6 (Figure 7K), CX3CL1 (Figure 7L), ANG-2 (Figure 7M), and soluble TNFR1 (Figure 7N). These data further corroborate our findings in the Cedars-Sinai and Northwestern cohorts and demonstrate that EPX positively correlates with inflammation in hospitalized ICU patients.

## Discussion

The practice of prescribing antibiotics for viral infections is a widespread problem (8–10, 37). Our study highlights the pernicious effects of antibiotic use during viral infections and defines a mechanism whereby antibiotics perturb the gut mycobiome and result in lung eosinophilia. In turn, lung eosinophils, via release of MBP-1, suppress alveolar macrophage clearance of bacteria. Clinical evidence also demonstrated that eosinophil levels positively correlate with antibiotic use, systemic inflammation, and ICU LOS. Altogether, our work indicates that antibiotic use may be counterproductive during influenza infection and attenuate antibacterial immunity.

Microaspiration of nasopharyngeal secretions into the lungs is a common occurrence in healthy individuals, but infections rarely occur as the contents are rapidly cleared by immunologic mechanisms that have evolved within the nonsterile airspaces (38, 39). However, respiratory viral infections impair host defense mechanisms, thereby providing aspirated commensal flora an opportunity to establish an infection (40). In fact, most bacterial pneumonias are precipitated by an antecedent viral infection (41). Therefore, common causes of pneumonia are, predictably, nasal commensals such as *S.* *aureus* and *S.* *pneumoniae* (40). Moreover, critically ill patients with *S.* *aureus* colonization have a 15-fold increased risk of developing pneumonia (42).

Development of bacterial pneumonia after an influenza infection is due to a complex host-pathogen interrelation that includes synergy between bacterial and viral virulence factors and disruption of host immunologic functions that normally prevent bacteria growth in the lower respiratory tract (43). Our data corroborate this interaction, as the antibiotic-driven effect in worsening lung injury occurred only in the coinfection model and was not seen in the single-injury models with influenza or MRSA alone. Moreover, the single-injury model with MRSA had clearance of bacteria from the lungs by the second day after infection, whereas in the 2-hit model, MRSA was still persistent at high levels 2 days after bacterial challenge. A preceding influenza infection primes the lung microenvironment to be more susceptible to bacterial superinfection (44). Indeed, influenza infection attenuates alveolar macrophage numbers and disrupts several immunologic pathways required for bacterial recognition (18, 19, 45, 46). Our work suggests that antibiotic treatment during influenza infection confers an additional risk that further attenuates effective macrophage clearance of bacteria.

Antibiotics have beneficial effects in helping eradicate bacterial infections, but inappropriate use can also have negative consequences. Antibiotics increase the risk of bloodstream infections by gut bacteria during SARS-CoV-2 infection (13). The current work further demonstrates the deleterious effects of antibiotics on lung host defense. Teleologically, eosinophils have a role in antiviral immunity and are likely recruited to the lung in that capacity during influenza infection (47–49). However, our findings demonstrate that eosinophils have detrimental immunologic consequences by suppressing macrophage antibacterial immunity mediated via the secretion of MBP-1. Accordingly, eosinophil recruitment may represent a homeostatic role in facilitating resolution of inflammation and repair during influenza infection but have harmful bystander effects on host immunity necessary for the clearance of aspirated bacteria.

Eosinophils are commonly associated with type 2 inflammation such as that found with allergic and helminthic diseases (50). However, the current work defines a role for eosinophils in negatively regulating lung immunity. Peripheral eosinophil levels are higher in those with severe COVID-19 compared with those with moderate illness (51), which is consistent with our findings that eosinophils may have detrimental immunologic consequences during viral illness. Eosinophilia is a common occurrence in patients receiving antibiotics (52). Though it is often presumed to be secondary to a drug reaction, we provide evidence that antibiotic-induced fungal dysbiosis drives the increase in lung eosinophils. We also found eosinophil levels correlated with the hospital LOS for influenza patients and ICU LOS for patients requiring mechanical ventilation. Additionally, plasma EPX levels were higher in those with ARDS compared with those at risk, which is consistent with the fact that the intensity of EPX immunostaining is increased in the lungs of patients with ARDS compared with control (53). Elevated eosinophils in the BAL have been associated with worse outcomes in lung transplant patients and are notably associated with fungal infection (54). Several studies in experimental murine models of lung fibrosis have also found that eosinophils are profibrotic (55–57). Our study therefore adds to the growing evidence that eosinophils have harmful effects that promote nonallergic inflammation in both acute and chronic lung diseases.

The current practice guidelines recommend that empiric antibiotics should be started for those presenting with symptoms consistent with a community-acquired pneumonia but should be promptly discontinued if a respiratory viral infection (e.g., influenza, SARS-CoV-2) is identified (58). Without question, antibiotics serve a purpose in treating bacterial infections, but their misuse when no bacterial infection exists may increase health risks to both the individual and the general public. The current study identifies a paradoxical effect of antibiotic use during influenza infection whereby lung defenses have diminished capacity to defend against a subsequent MRSA challenge. Influenza infection primes the lungs to be more susceptible to a subsequent bacterial infection, and our data suggest that antibiotic-induced eosinophilia further exacerbates the risks. This study defines a mechanism by which antibiotic use during influenza infection alters lung innate immunity and challenges the common clinical practice of prescribing antibiotics during influenza when there is no evidence of bacterial infection.

## Methods

### Animal experiments and sex as a biological variable.

EoCre mice, transgenic mice previously characterized to express Cre recombinase in eosinophils, were provided by Elizabeth Jacobsen (Mayo Clinic, Phoenix, Arizona, USA) (59). The EoCre mice were crossed with ROSA^mTmG^ transgenic mice (The Jackson Laboratory, strain 007676) to create transgenic mice that express GFP in eosinophils and tdTomato in all other cell types.

For the 2-hit influenza-MRSA model, mice were fed water ad libitum, either control water containing sucrose (50 g/L) or an antibiotic cocktail containing vancomycin (0.5 g/L), neomycin (1 g/L), ampicillin (1 g/L), and metronidazole (1 g/L) (VNAM; MilliporeSigma) and sucrose (50 g/L), for 14 days (starting day –7 and ending day 7). Water bottles were changed twice a week. On day 0 (7 days after starting VNAM), animals were intranasally infected with mouse-adapted H1N1 influenza virus A/Puerto Rico/8/34 (PR8) (250–500 PFU in 25 μL of PBS) under inhalation anesthesia (isoflurane, Piramal Critical Care Inc.). On day 7, all groups were switched to control water containing sucrose (50 g/L). Animals were infected with MRSA (strain USA300) or *S.* *pneumoniae* (serotype 19F) on day 10 by administration of 1 × 10^7^ CFU per mouse or 1 × 10^6^ CFU per mouse, respectively, in 40 μL of PBS intratracheally. Mice were sacrificed on the indicated days after PR8 infection for tissue collection. In some experiments, an additional treatment arm that included an antifungal drug with VNAM treatment in the drinking water was evaluated by addition of fluconazole (0.5 g/L) or amphotericin (0.1 g/L). Other studies added intraperitoneal injection of an isotype (100 μg/mouse; Bio X Cell, clone HRPN; BE0088) or anti–IL-5 antibody (100 μg/mouse; Bio X Cell, clone TRFK5; BE0198).

All mice were on the C57BL/6 background and were used for experiments between the ages of 8 and 12 weeks. In the 2-hit experiments, pilot experiments identified a similar phenotype in both male and female mice. Although no significant difference was identified in the effect between sexes, most of the subsequent studies were performed predominantly in female mice to reduce variability. For experiments in which eosinophils were isolated from transgenic mice, both male and female mice were used.

### Human studies.

The Research Informatics and Scientific Computing Core at Cedars-Sinai Medical Center (CSMC) through the Honest Enterprise Research Broker committee provided clinical data from patients admitted for influenza infection in 2019. Deidentified data were provided by investigators at Northwestern University through an established data use agreement. Deidentified samples and associated metadata were provided by investigators at the University of Pittsburgh Medical Center through a material transfer agreement.

Cohort 1 included clinical data collected from all patients admitted in 2019 to CSMC with the diagnosis of influenza infection. The eosinophil level at admission and the last measured value prior to discharge were evaluated in those that were treated with antibiotics for more than 4 days during the hospitalization. Furthermore, the mean eosinophil level of all measurements during the hospitalization was correlated with the hospital LOS.

Cohort 2 examined the pattern of serum absolute eosinophils and BAL percentage eosinophils using deidentified data from the SCRIPT study, a single-site prospective cohort study of patients hospitalized at Northwestern Memorial Hospital ICU who required mechanical ventilation and underwent a BAL for suspected pneumonia (17, 60). We used a day-by-day map prepared for the *CarpeDiem* project (60). If a patient had multiple eosinophil values measured on one day, the mean value for that day was used for analysis. We removed patients who had received steroids during their ICU course, leaving a cohort of 200 patients who underwent 345 BALs during 2,487 ICU days. For patients with multiple ICU stays, we took data only from their first ICU stay for analysis.

Cohort 3 used deidentified plasma (*n* = 120) and metadata from ICU patients on mechanical ventilation at the University of Pittsburgh Medical Center (61). Plasma from ICU patients was diluted 1:25 in PBS, and duplicate measurements of EPX were determined with the Human Eosinophil Peroxidase (EPX) ELISA kit (MyBioSource, MBS2024555) following the manufacturer’s protocols. Host-response plasma biomarkers from this patient cohort were measured with a custom-made Luminex panel (R&D Systems) as previously described (61).

### Bronchoalveolar lavage.

BAL fluid from mice was collected by cannulation of the trachea with an 18-gauge angiocath (BD, 381134) with the lungs in situ followed by instillation of 1 mL of sterile PBS and immediate suctioning to maximally recover the instillate. The instillation and recovery process were repeated 2 more times (total 3 mL). The BAL fluid was counted with an automated cell counter (Bio-Rad Laboratories, TC20) and centrifuged (1,500 rpm [277*g*] for 10 minutes). The supernatant was separated from the cell pellet and frozen at –80°C until use. The cell pellet was immunostained for flow cytometry analysis to quantify immune subpopulations.

### Lung cell dissociation.

Mouse lungs were placed in a Petri dish and washed with PBS to remove remaining blood. Cells were dissociated using the Lung Dissociation Kit, mouse (Miltenyi Biotec, 130-095-927), in conjunction with gentleMACS Dissociators (Miltenyi Biotec) following the manufacturer’s protocol. The cell suspension was centrifuged at 300*g* at 4°C for 10 minutes, and the cell pellet was resuspended in 3 mL of RBC lysis buffer (eBioscience, 00-4333-57). After 1 minute at room temperature, 10 mL of PBS was added to neutralize the RBC lysis buffer and centrifuged at 300*g* at 4°C for 10 minutes. The supernatant was removed, and the cell pellet was washed one more time with 10 mL of PBS. The cells were resuspended in cold flow buffer (PBS plus 5% FBS), counted, and processed for flow cytometry.

### Flow cytometry.

Single-cell suspensions were stained with a mixture of fluorochrome-conjugated antibodies (1:100 dilution) for 30 minutes at 4°C. After incubation, cells were washed with flow buffer (PBS plus 5% FBS), fixed with 4% paraformaldehyde (10 minutes at room temperature), washed, and stored in flow buffer until flow cytometry analysis. Data were acquired by a BD LSRFortessa Cell analyzer and analyzed with FlowJo version 9 (BD). Flow antibodies are listed in Supplemental Table 7.

### Protein assays.

Total protein in mouse BAL fluid was measured by BCA assay (Thermo Fisher Scientific, 23225), and ELISAs for murine IFN-γ (R&D Systems, DY485-05), IL-1β (R&D Systems, DY401-05), and PRG2 (MBP-1; MyBioSource, MBS2022666) were performed per manufacturer’s protocol.

### Immunofluorescent assays.

Murine lungs were inflated with 10% neutral formalin and then placed in 70% alcohol after 24 hours. Tissues were processed for paraffin embedding, and then formaldehyde-fixed, paraffin-embedded (FFPE) tissues were cut into 7-μm-thick sections. Slides underwent 1× citrate buffer antigen retrieval and permeabilization with 10% methanol and 0.4 H_2_O_2_ and were blocked with 5% BSA. Lung sections were stained with anti-GFP rabbit polyclonal antibody (1:100; Rockland, 600-401-215) followed by donkey anti-rabbit–Alexa Fluor 488 antibody (1:500; BioLegend, 406416) and DAPI counterstain (1:500; Invitrogen, D1306).

Human lungs were received from the Department of Pathology at CSMC. FFPE tissues were cut into 10-μm-thick sections and processed for immunostaining for MBP-1 (PRG2) as above using a rabbit anti-PRG2 antibody (1:500; Invitrogen, PA5-102628) followed by donkey anti-rabbit–Alexa Fluor 488 antibody (1:500; BioLegend, 406416) and DAPI counterstain (1:500; Invitrogen, D1306). All immunofluorescence images were obtained with a Nikon Ti2 microscope.

### Eosinophil isolation and conditioned medium collection.

Bone marrow cells were collected from the femora of wild-type EoCre-mTmG transgenic mice. Femora were isolated, and both ends of bones were cut off. The shaft of the femur with the patellar end downward was then placed into a 0.5 mL microcentrifuge tube with a small hole on the bottom that was nested in a 1.5 mL microcentrifuge tube. The bones were centrifuged at 18,000*g* for 30 seconds. The supernatant was decanted, and the bone marrow pellet was resuspended in 500 μL of PBS and transferred to a 15 mL conical vial. The bone marrow suspension was centrifuged at 300*g* at 4°C for 8 minutes. Afterward, the supernatant was aspirated, and the bone marrow pellet was resuspended in 500 μL of RBC lysis buffer. After 1 minute at room temperature, 10 mL of PBS was added to neutralize the RBC lysis buffer. The bone marrow suspension was centrifuged at 300*g* at 4°C for 8 minutes and, after removal of the supernatant, was resuspended in 200 μL of chilled sorting buffer (0.5 M EDTA, 2% BSA in HBSS). Eosinophils were isolated with a BD Influx System by sorting for GFP^+^, RFP^–^ cells (Supplemental Figure 13). Purified eosinophils were centrifuged at 400*g* for 5 minutes at 4°C and resuspended in IMDM plus 10% FBS without antibiotic at a concentration of 5 × 10^4^ cells per 250 μL. Eosinophils were either immediately used for bacterial killing assays or cultured at 37°C for 24 hours to collect the conditioned medium, which was frozen at –80°C until required for further experiments.

### Bacterial culture.

Frozen bacterial stock (MRSA, USA 300) was directly scraped with an inoculation loop, added to 3–4 mL of tryptic soy broth (TSB; VWR, 470177-386) in a 15 mL tube, and incubated overnight at 37°C with shaking at 200 rpm. After an overnight incubation, the bacterial culture was diluted 1:25 in a 96-well plate (Greiner Bio-One, 655180) and quantitated by measurement of the amount of absorbance at 550 nm. MRSA was diluted in 3 mL of TSB and subcultured for 2 hours and 45 minutes with shaking at 200 rpm at 37°C to reach the middle of exponential growth. The subculture was then diluted 1:5 in TSB and quantified by measurement of the amount of absorbance at 550 nm. The amount of MRSA used in subsequent assays was washed twice with PBS and resuspended in PBS at the appropriate concentration. *S.* *pneumoniae* were similarly grown but in Brain Heart Infusion Broth medium (Thermo Fisher Scientific, R060270) and cultured at 37°C in 5% CO_2_.

### Bacterial colony-forming units.

To determine CFU of bacteria, 100 μL of conditioned medium from in vitro bacterial killing assay or lung homogenates (100 mg/mL in PBS) was used as a starting point. Serial 1:10 dilutions were then created with PBS for a total of 4 concentrations including the original stock. An agar plate containing 5% sheep blood (BD Trypticase Soy Agar II, BD Biosciences 221261) had 4 quadrants demarcated on the plate, and 10 μL of each dilution was streaked out in respective quadrants using sterile loops (Celltreat/Fisher, 229612). Blood agar plates were incubated at 37°C until individual colonies were clearly visible.

Fiji software (ImageJ2 version 2.14.0/1.54F) was used to count the CFU as follows. Individual JPEG images of each quadrant were converted to 16 bit and had the threshold adjusted to highlight distinct colonies while eliminating background noise. A watershed function was used to separate individual colonies that may have overlapping borders after thresholding (Process → Binary → Watershed). Subsequently, individual colonies could be counted using the analyze particle function (Analyze → Analyze Particle).

### In vitro bacterial killing assay and phagocytosis assay.

RAW 264.7 cells (ATCC, TIB-71) were plated in a 48-well plate at 5 × 10^4^ cells per well in 250 μL of DMEM (Corning, 10-013-CV) or IMDM (Gibco, 31980030) plus 10% FBS (Cytiva, 16777-014) without antibiotic and incubated at 37°C. The next day, RAW 264.7 cells were washed, and MRSA (200 CFU in 250 μL of DMEM 10% FBS) was added. In some conditions, 5 × 10^4^ eosinophils were added to the culture. Alternatively, 250 μL of conditioned medium from eosinophil cultures was used. All conditions were cultured at 37°C with 5% CO_2_ for 4 hours, at which point conditioned medium was collected and placed on ice for immediate determination of CFU. In parallel experiments, RAW 264.7 cells were cultured at 37°C with 5% CO_2_, and either BSA (1 μg/mL; control) or recombinant MBP-1 (1 μg/mL; Cedarlane, RPB650MU02-2) was added for 2 hours before CFU determination.

RAW 264.7 cells (10^4^ cells per well) were cultured in 96-well plates for 48 hours before addition of pHrodo Red *S.* *aureus* bioparticles (Thermo Fisher Scientific, A10010). Phagocytosis was evaluated by real-time immunofluorescent imaging of wells cultured at 37°C and 5% CO_2_ (Incucyte Sartorius). Some conditions had addition of BSA (1 μg/mL) or recombinant MBP-1 (1 μg/mL) or were replaced with eosinophil conditioned medium. Every hour, images of the whole well were captured using a ×4 objective lens with a 400-millisecond acquisition time for the red channel. Images underwent phase contrast masking to distinguish cells from the background during analysis. Red channel background noise was minimized using top-hat segmentation with a 100 μm radius, and a threshold of 1 or 2 was applied for the red channel. The internalization of bioparticles or heat-killed MRSA was measured using a mask for red-positive cells. Phagocytosis was quantified using total red fluorescence intensity normalized by confluence and intensity at time 0.

For in vivo phagocytosis by alveolar macrophages, pHrodo Red *S.* *aureus* bioparticles were diluted in sterile PBS (1 part bioparticle and 4 parts PBS). Additionally, BSA or recombinant MBP-1 was added to the solution to make a final concentration of 1 μg/mL. Mice had 50 μL of the solution intranasally instilled into the lungs. After 1 hour, cells were lavaged from the lungs, and phagocytosis was determined by the percentage of alveolar macrophages (CD45^+^, CD11c^+^, Siglec-F^+^) that acquired pHrodo signal by flow cytometry (Supplemental Figure 14).

Precision-cut lung slice (PCLS) cultures were prepared from unchallenged euthanized mice. The lungs were inflated with 1 mL of 2% low-melting-point agarose solution heated at 37°C. The inflated lungs were embedded in low-melting-point agarose, allowed to solidify on ice, and then sectioned into 300-μm-thick slices using a vibratome. The lung slices were placed into 12-well plates containing prewarmed DMEM-F12 medium without phenol red and cultured at 37°C with 5% CO_2_ for 24 hours. The next day, the agarose was removed from each PCLS by washing once with PBS. Subsequently, 300 μL of DMEM without antibiotics supplemented with 1 μg/mL of recombinant MBP-1 or BSA was added. Additionally, pHrodo Red *S.* *aureus* bioparticles (1:10 final dilution) were added to all conditions. After a 24-hour culture, the PCLS was washed once with PBS and then fixed with 4% paraformaldehyde for 30 minutes at room temperature. After another wash, the slices were placed on slides, mounted with mounting medium, and then stored at 4°C. Images were taken with a Zeiss LSM780 confocal microscope, and number of red fluorescent particles was quantified per each ×10 field. Multiple random fields were imaged and averaged together for the phagocytosis measurement.

### Single-cell RNA-Seq and data processing.

Lungs were isolated and single-cell suspensions were processed for scRNA-Seq as previously described (62). In brief, control (*n* = 4) and VNAM-treated (*n* = 4) mice were sacrificed at day 10 after PR8 infection, and lungs were removed and enzymatically digested with elastase and lipase/DNase. Cell suspensions were processed for single-cell capture and library preparation (10x Genomics Single Cell 3′ Reagent Kits) and sequenced on the Illumina NovaSeq 6000.

CellRanger v7.0.0 software (10x Genomics) was used with the default settings for demultiplexing, aligning reads with STAR software (10x Genomics), and counting unique molecular identifiers. Cell hashing data were demultiplexed using the CellRanger default setting. The single-cell analysis R package Seurat v5 was used for further data analysis (63). For quality control and filtering out of low-quality cells, only cells expressing more than 500 genes (defined as genes detected in at least 3 cells) and fewer than 10% mitochondrial genes were selected. To minimize doublet contamination, doublets were removed using DoubletFinder (64), and doublet ratio was estimated using a fit model generated from the suggested “multiplet rate”/“number of cells recovered” ratio, as stated in the 10x Genomics user manual (65).

We used default normalization and data scaling procedures from the Seurat package, which involve log normalization and linear model for data scaling. For data integration, we used the batch correction package Harmony (66). Principal component analysis was performed using the 3,000 most variable genes, and the first 30 independent components were used for downstream unbiased clustering with a resolution of 0.6. The uniform manifold approximation and projection (UMAP) method was used for unsupervised clustering visualization. The cell cluster identities were determined using known gene markers of lung cell types (67). To identify differentially expressed genes (DEGs) between different clusters and groups, we used the default differential expression testing based on the nonparametric Wilcoxon’s rank sum test by Seurat. Genes with adjusted *P* less than 0.01 were input into Ingenuity Pathway Analysis (IPA; QIAGEN) to evaluate regulatory pathways.

### RNA-Seq and data processing.

RAW 264.7 cells were seeded in 12-well plates at a density of 5 × 10^5^ cells per well in 500 μL of DMEM supplemented with 10% FBS and incubated at 37°C. The next day, RAW 264.7 cells were rinsed once with PBS, and 500 μL of eosinophil conditioned medium or control epithelial conditioned medium (MLE-15 cells cultured at 5 × 10^4^ cells per 250 μL for 24 hours) was added. In parallel experiments, RAW 264.7 cells were incubated in eosinophil conditioned medium with the addition of an isotype antibody (2 μg/mL; BioLegend, Poly29018) or anti–MBP-1 antibody (2 μg/mL; Thermo Fisher Scientific, PA5-102628). After 24 hours of culture, cells were lysed for RNA extraction using the RNeasy Mini Kit (QIAGEN, 74104), and the extracted RNA was stored at –80°C before RNA sequencing with NovaSeq 6000.

Raw reads were aligned with STAR aligner 2.7.1 (68) with default parameters, using a mouse mm10 reference genome downloaded from iGenomes (https://support.illumina.com/sequencing/sequencing_software/igenome.html). Raw counts were generated with featureCounts 2.0.3 (10x Genomics). DEGs were generated using DESeq2 2.11.40.8 (10x Genomics) with raw counts. These processes were performed using Galaxy server (usegalaxy.org). A normalized transcripts per million (TPM) file was generated using the R package. DEGs were input into IPA, and genes with an adjusted *P* less than 0.01 were used for Disease and Function Analysis.

### Stool DNA isolation, microbiome sequencing, and analysis.

DNA was extracted from mouse stool using a modified QIAamp DNA Mini Kit (QIAGEN, 51306). Stool was mixed with lysis buffer and homogenized with 0.1 mm and 0.5 mm glass beads (Biospec, 11079101z and 11079105z, respectively) using a bead beater at high speed for 1 minute. Tubes were centrifuged for 5 minutes, and the supernatant was decanted. Stool was resuspended in 500 μL stool DNA stabilizer (B Bridge International, 1038111100) and homogenized with glass beads using a bead beater at high speed for 1 minute. Tubes were then heated at 95°C for 5 minutes, subjected to a bead beater again for 1 minute, heated at 95°C for 5 minutes, then vortexed for 5 seconds. Next, 300 μL Buffer AL (Qiagen, 51306) and 22 μL proteinase K were added, vortexed for 30 seconds, and incubated at 70°C for 10 minutes. Subsequently, 600 μL phenol:chloroform:isoamyl alcohol (Thermo Fisher Scientific) was added, and the tubes were subjected to a bead beater for 30 seconds. Tubes were centrifuged for 5 minutes at 16,000*g*, and the upper aqueous phase was removed and transferred to a new tube to which 700 μL of ethanol was added and mixed. Solution was added to QIAamp collection tubes, and the kit protocol for DNA extraction was followed.

Stool bacteria burden and fungus burden were measured by PCR quantification using 16S primers (forward: ACTCCTACGGGAGGCAGCAGT; reverse: ATTACCGCGGCTGCTGGC) and FungiQuant (69) primers (forward: GGRAAACTCACCAGGTCCAG; reverse: GSWCTATCCCCAKCACGA), respectively. Stool was collected from mice subjected to the 2-hit injury at days –7, 0, 10, and 12 to evaluate changes in α- and β-diversity. ITS sequencing was repeated for stool collected at day 10 to evaluate changes in the relative abundance at the family and genus level.

Bacterial 16S rRNA and fungal ITS rRNA gene amplicons were generated using high-fidelity Platinum SuperFi Polymerase (Life Technologies). The following primers were used (underlined regions indicate the Illumina adapter sequence and boldface regions indicate the primer sequence): (a) bacterial 16S rRNA primers 8F (TCGTCGGCAGCGTCAGATGTGTATAAGAGACAG**AGAGTTTGATCMTGGCTCAG**) and R357 (GTCTCGTGGGCTCGGAGATGTGTATAAGAGACAG**CTGCTGCCTYCCGTA**) with annealing temperature 48°C and 25 PCR cycles; (b) fungal ITS rRNA primers ITS1f (TCGTCGGCAGCGTCAGATGTGTATAAGAGACAG**CTTGGTCATTTAGAGGAAGTAA**) and ITS2 (GTCTCGTGGGCTCGGAGATGTGTATAAGAGACAG**GCTGCGTTCTTCATCGATGC**) with annealing temperature 48°C and 35 PCR cycles. PCR reactions were purified using the ZR-96 DNA Clean-up Kit (Zymo Research). Amplicons were qualified on the 4200 TapeStation using the Agilent D1000 ScreenTape System (Agilent Technologies).

Amplicons from up to 384 samples were uniquely indexed in a PCR reaction using Nextera XT Index Kit v2 Sets A–D (Illumina) and KAPA HiFi HotStart ReadyMix (Roche). Library enrichment was performed using 8 cycles of PCR. Multiplexed samples were pooled at equal volume by library type. The pooled libraries were purified using HighPrep PCR Magnetic Beads (MagBio Genomics) and subsequently assayed on a 4200 TapeStation to check final sizing. Samples were sequenced on the MiSeq platform (Illumina) with paired-end 300 bp sequencing chemistry. Raw data processing and run demultiplexing were performed using on-instrument analytics per manufacturer recommendations. 16S (V1–V2 region) and ITS1 sequencing of mouse stool samples was performed as previously described (70).

### Statistics.

All experiments had a minimum of 3 independent biological replicates performed. Technical replicates were averaged to represent 1 independent sample. Statistical analysis (2-sided) and figure generation were performed with Prism version 10 (GraphPad Software). Statistical tests are listed within the corresponding figure legends. A *P* value of less than 0.05 was considered statistically significant.

### Study approval.

All animal experiments were approved by the IACUC (IACUC009018) at CSMC. Data collected from patients admitted for influenza infection at CSMC were approved by the Institutional Review Board (STUDY00000219). Data from Northwestern University and samples collected at the University of Pittsburgh were approved by the IRB at their respective institutions, and all data and samples sent to CSMC were deidentified.

### Data availability.

The scRNA-Seq data can be obtained from the NCBI’s Gene Expression Omnibus (GEO) database (GSE243542). RNA-Seq data can be obtained from the GEO database (GSE259429). Values for all data points in graphs are reported in the Supporting Data Values file.

## Author contributions

MSSRZ, TP, SAB, SD, DMU, and PC conceived or designed the study. MSSRZ, TP, SAB, KL, CAG, RGW, GDK, AM, YZ, BJM, MEM, AMM, CY, BRS, CMS, YW, KM, SC, BLV, IVC, SG, SLC, FSS, SD, DMU, and PC acquired, analyzed, or interpreted data. MSSRZ, TP, and PC drafted the work or substantively revised it. The order of MSSRZ, TP, and SAB as co–first authors was assigned by the extent of the work performed by the individual for this study.

## Supplementary Material

Supplemental data

Supplemental table 1

Supplemental table 2

Supplemental table 3

Supplemental table 4

Supplemental table 5

Supplemental table 6

Supplemental table 7

Supporting data values

## Version 1 09/10/2024

In-Press Preview

## Version 2 11/01/2024

Electronic publication
