# Supplementary material for: Antibiotic use during influenza infection augments lung eosinophils that impair immunity against secondary bacterial pneumonia
Source: J Clin Invest. 2024 Sep 10;134(21):e180986. doi: 10.1172/JCI180986 (PMC11527449; doi:10.1172/JCI180986)
Supplement: Supplemental data [file jci-134-180986-s058.pdf]

Supplemental Figures

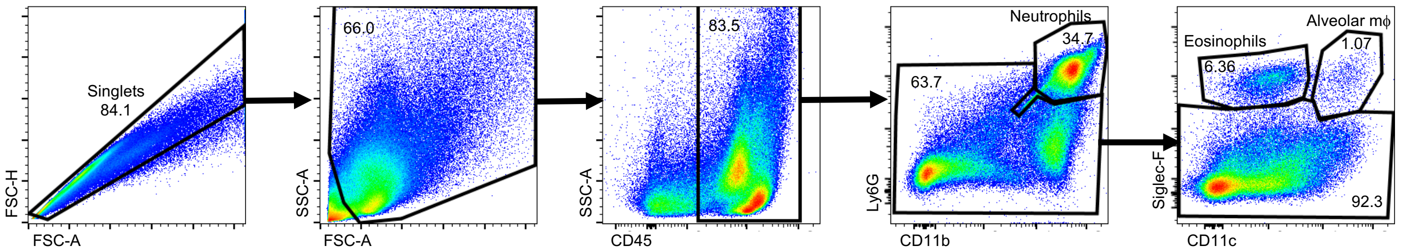

Supplemental Figure 1. Flow gating strategy for BAL immune cells. BAL cells were immunostained for flow cytometry quantification of cell subsets.

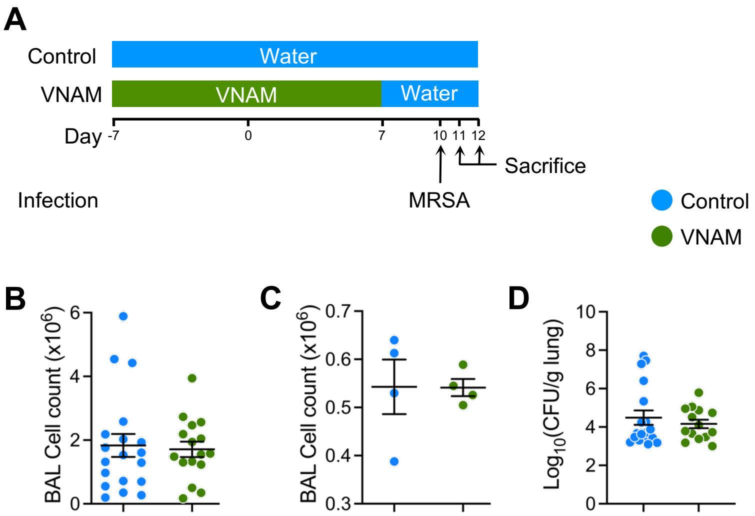

Supplemental Figure 2. Antibiotic treatment of mice has no effect on MRSA infection. A) Mice were treated with control and antibiotics (VNAM) for 14 days (day -7 to day 7) and then mice were given intranasal MRSA at day 10. Mice were sacrificed one and two days after infection (day 11 and 12) and evaluated for: **B)** BAL total cell count (day 11; n = 16-19) **C)** BAL total cell count (day 12; n = 4)), and **D)** CFU of bacteria (day 11 ; n = 14-18). No appreciable bacterial were found in the lungs two days after MRSA infection (day 12).

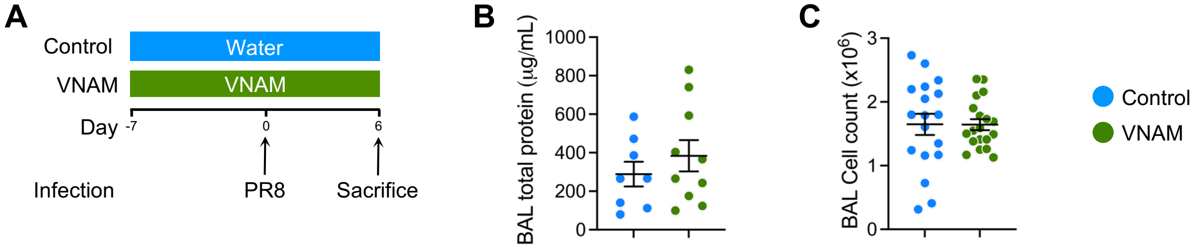

Supplemental Figure 3. Antibiotic treatment of mice has no effect on lung injury during influenza infection. A) Mice were infected with influenza (PR8, 250 PFU) at day 0. Control and antibiotics (VNAM) were started 7 days prior to PR8 infection to allow mice to equilibrate to the treatment prior to infection. Mice were sacrificed at day 6 after infection and evaluated for: **B)** BAL total protein (n = 8-10) and **C)** BAL total cell count (n = 18-19).

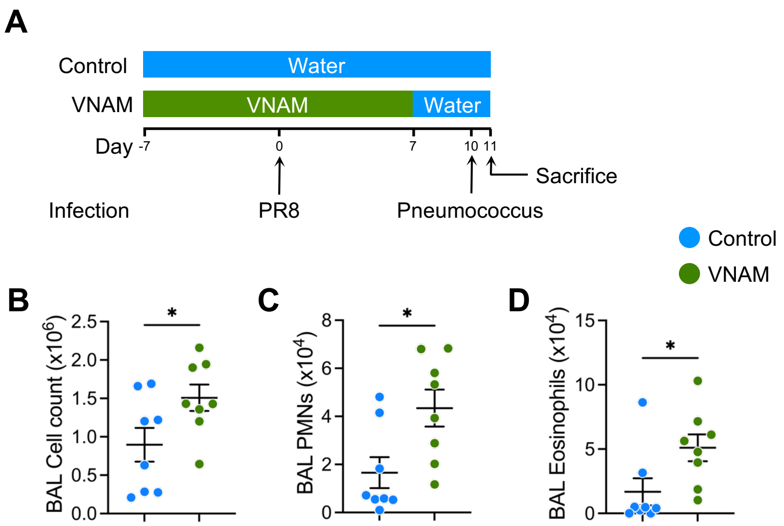

Supplemental Figure 4. Antibiotic treatment of mice during influenza infection augments lung inflammation after a subsequent challenge with *S. pneumonia*. A) Mice were infected with influenza (PR8, 250 PFU) at day 0. Control or antibiotics (VNAM) was started 7 days prior to PR8 infection to allow mice to equilibrate to the treatment and discontinued at day 7 to allow for it to wash out before pneumococcal challenge on day 10. Mice (n = 8) were sacrificed 1 day after bacterial infection, and BAL cells were quantified for **B)** total cell count, **C)** neutrophils, and **D)** eosinophils. *p<0.05 by Student's T-test.

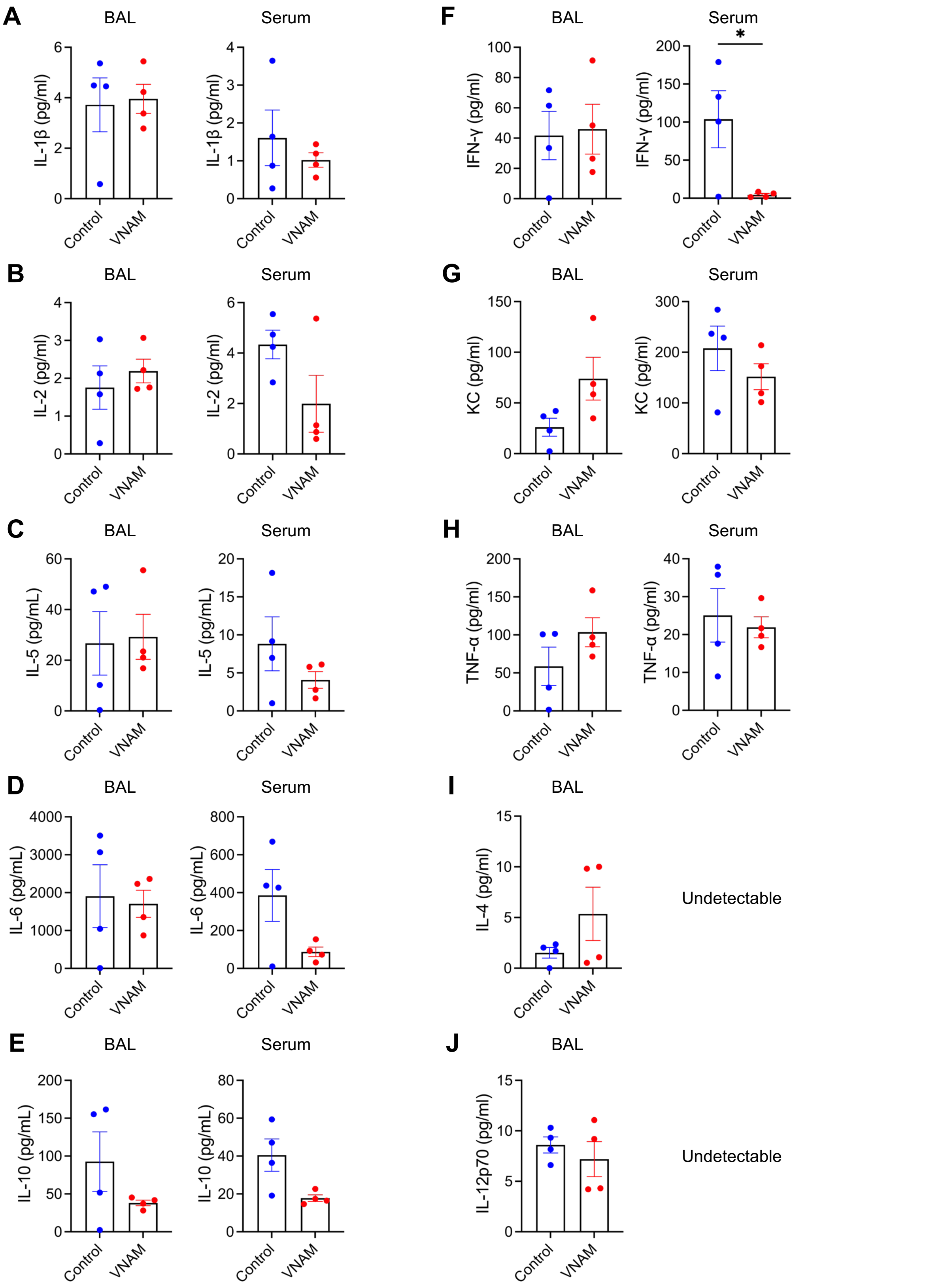

Supplemental Figure 5. Antibiotic treatment of mice has no effect on lung inflammation during influenza infection. Mice were infected with influenza (PR8, 250 PFU) at day 0. Control and antibiotics (VNAM) were started 7 days prior to PR8 infection to allow mice to equilibrate to the treatment and discontinued at day 7. Mice (n = 4) were sacrificed on day 10 for MesoScale evaluation of the BAL and serum for multiple cytokines: **A)** IL-1β, **B)** IL-2, **C)**, IL-5, **D)** IL-6, **E)** IL-10, **F)** IFN-γ, **G)** KC, **H)** TNF-α, **I)** IL-4, and **J)** IL-12p70.

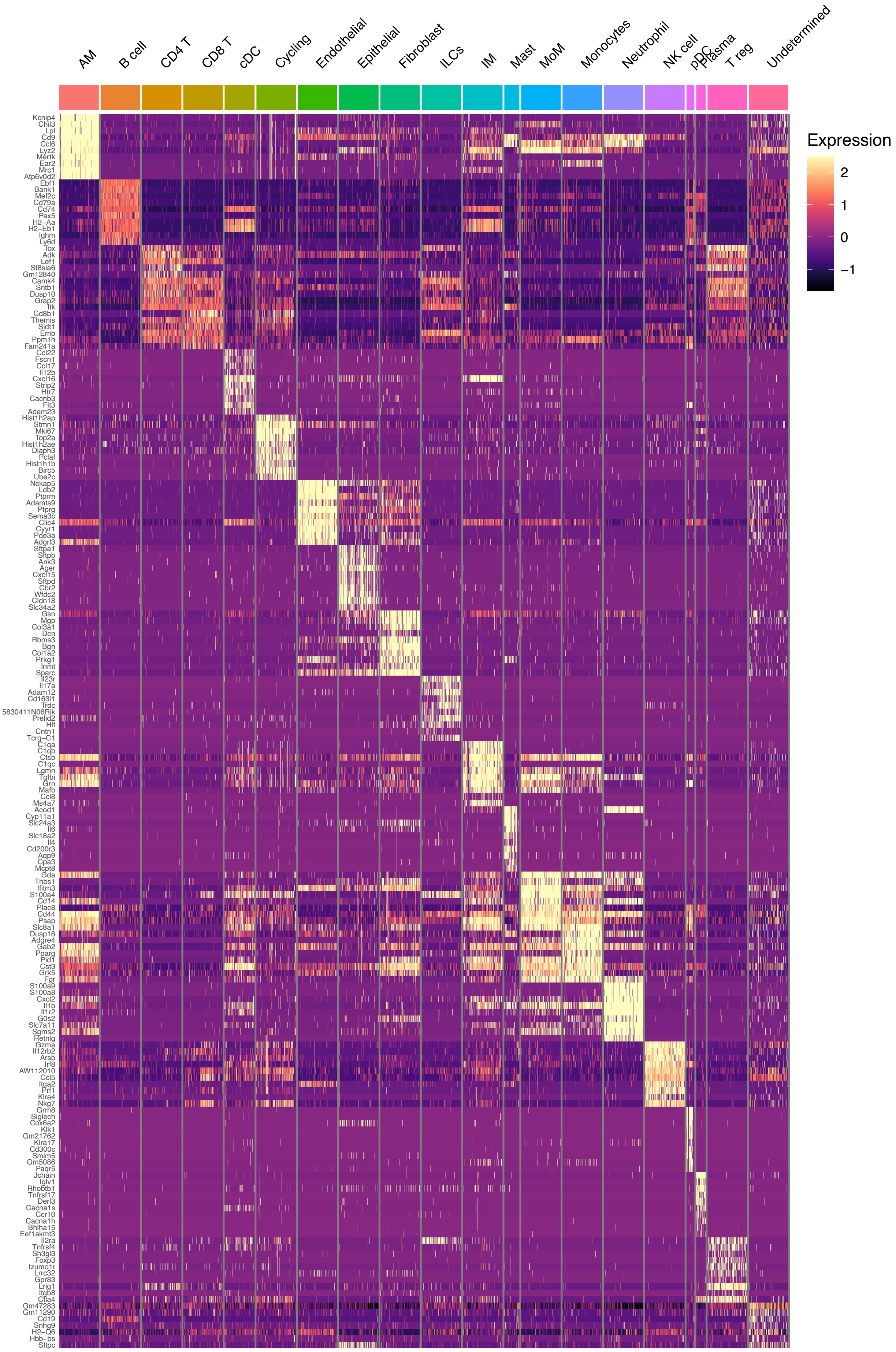

Supplemental Figure 6. Heatmap of the top 10 genes for each cell type in the scRNA-seq dataset.

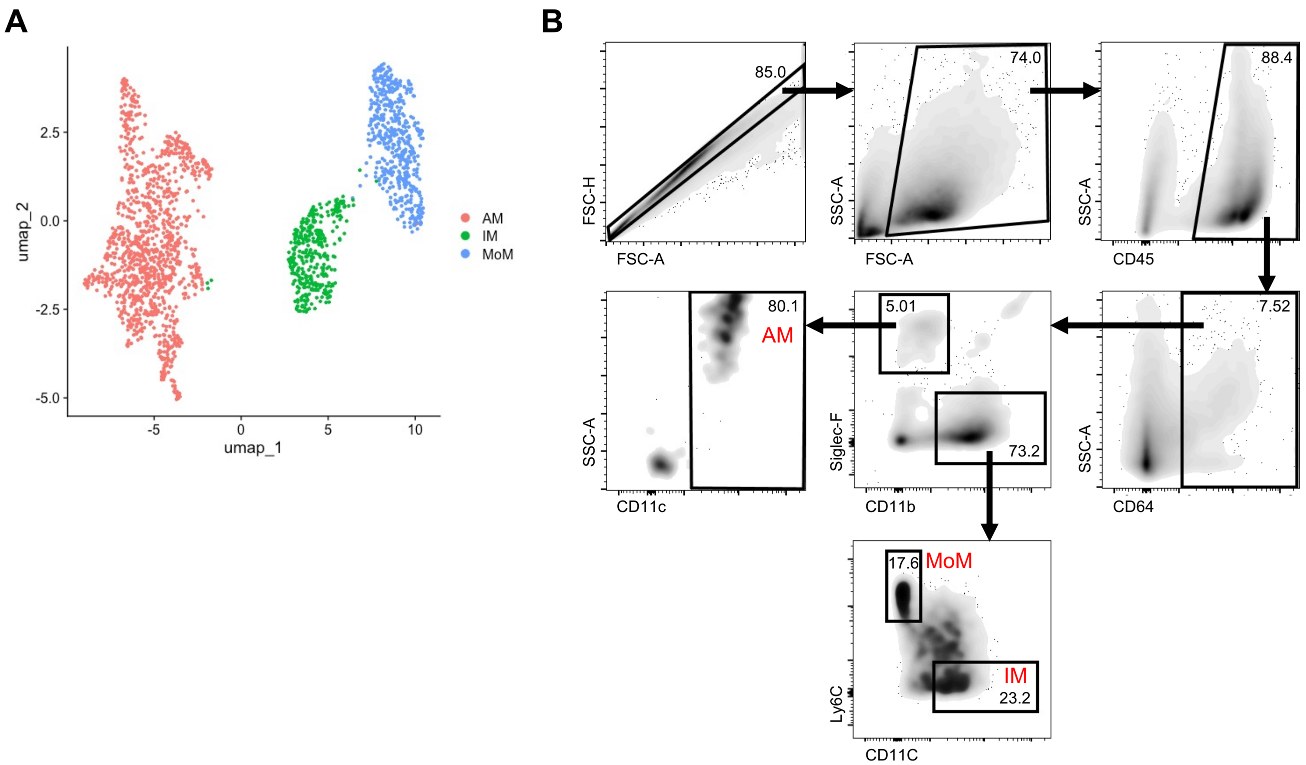

Supplemental Figure 7. Relative numbers of macrophage populations. A) Subclustering of the scRNA-seq data for alveolar macrophages (AM), interstitial macrophages (IM), and monocyte-derived macrophages (MoM). **B)** Flow cytometry gating strategy for macrophage populations in lung homogenates.

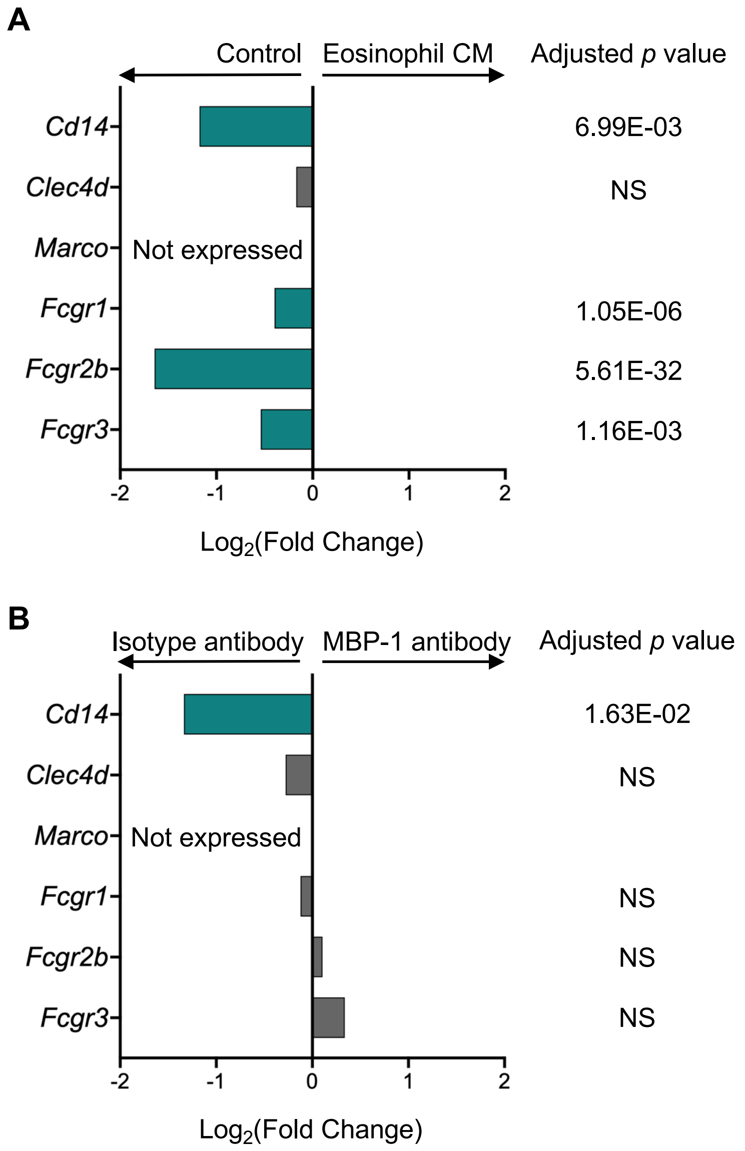

Supplemental Figure 8. Eosinophil conditioned medium suppresses macrophage phagocytosis receptors through the secretion of MBP-1. A) Macrophages were cultured in conditioned medium from either eosinophils or epithelial cell as a control (n =3). **B)** Macrophages were cultured in conditioned medium from eosinophils and with the addition of either an isotype antibody or anti-MBP-1 antibody (n = 3). Macrophages were processed for RNA-seq, and DEGs were determined between conditions (entire DEG list is provided in **Supplemental Table 3**). Relative fold-change values and the adjusted *p* value for phagocytosis receptors from the DEG list were presented in the graph.

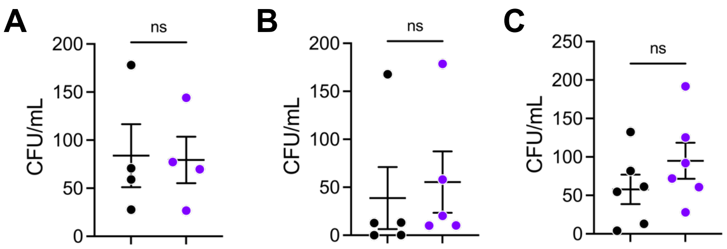

Supplemental Figure 9. Pretreatment of MRSA or macrophages with MBP-1 does not suppress MRSA growth. A-B) BSA (1 µg/mL) or recombinant MBP-1 (1 µg/mL) was added to **A)** Raw 264.7 cells or **B)** MRSA. After 1 hour, cells were washed with PBS, and then added to cultures. **A)** Pretreated Raw cells ($5x10^4$ cells) and 200 CFU of untreated MRSA were co-cultured in 48-well plate in 250 µL of antibiotic-free DMEM 10% FBS for 2 hours before determining CFU/mL of MRSA (n = 4). **B)** Untreated Raw cells ($5x10^4$ cells) and 200 CFU of pretreated MRSA were co-cultured in 48-well plate in 250 µL of antibiotic-free DMEM 10% FBS for 2 hours before determining CFU/mL of MRSA (n = 5). **C)** MRSA (200 CFU in 250 µL of DMEM 10% FBS) was cultured for 2 hours in the presence of either BSA (1 µg/mL) or recombinant MBP-1 (1 µg/mL) (n = 6).

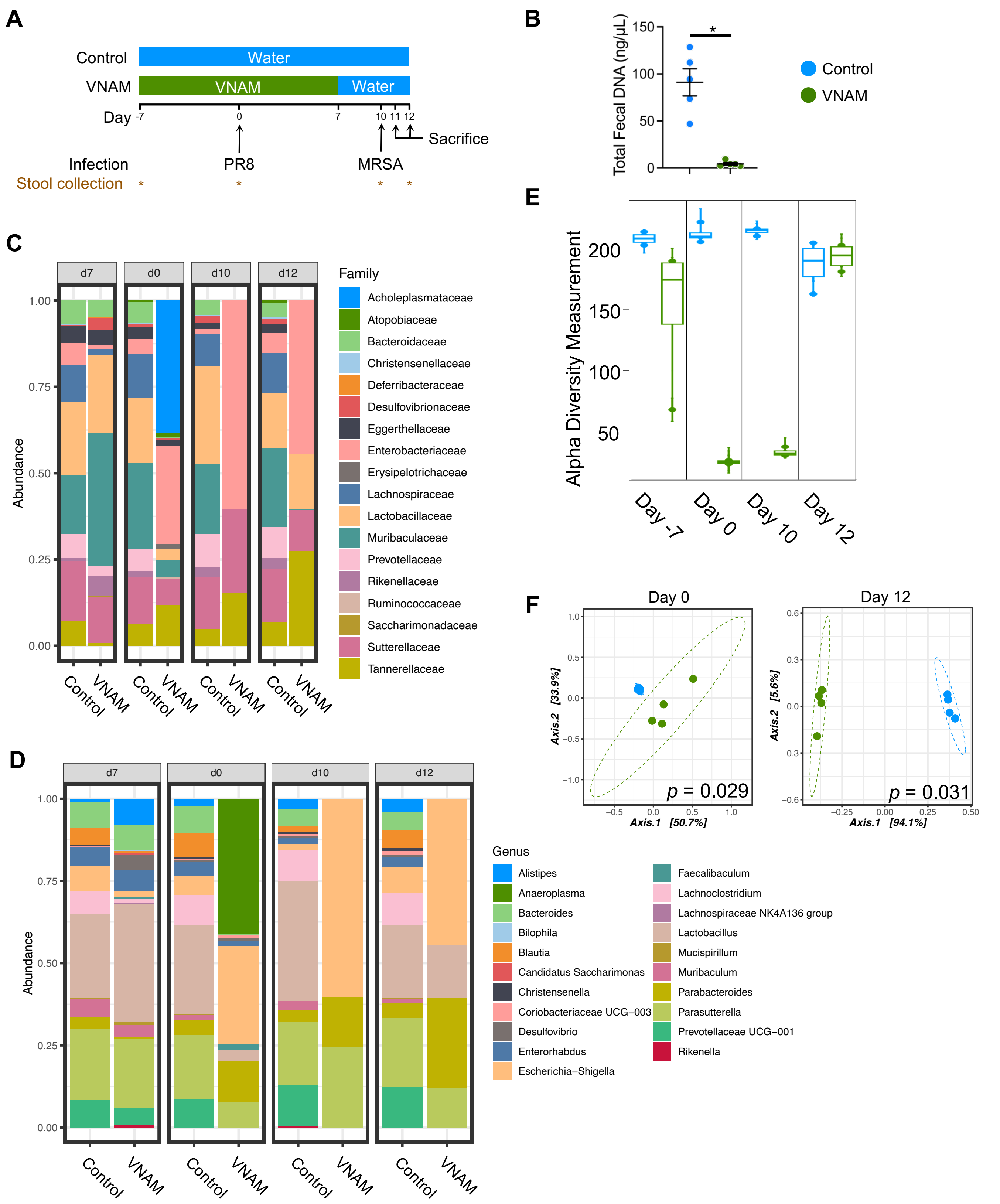

Supplemental Figure 10. Antibiotics causes dysbiosis during the influenza-MRSA two-hit challenge. A) Control and VNAM-treated mice infected with influenza (Day 0) followed by MRSA (Day 10) had stool collected for 16S sequencing (n = 4). **B)** 16S PCR demonstrates antibiotic depletion of bacteria from the gut microbiota after 7 days of treatment (n = 5). **p*<0.001 by Student's T-test. **C – D)** Mean relative abundance for control and VNAM groups at different timepoints during the two-hit model at the **C)** family and **D)** genus level. **E)** Chao index showed changes in alpha diversity. **F)** Principal coordinates analysis (PCoA) demonstrated changes in the beta diversity.

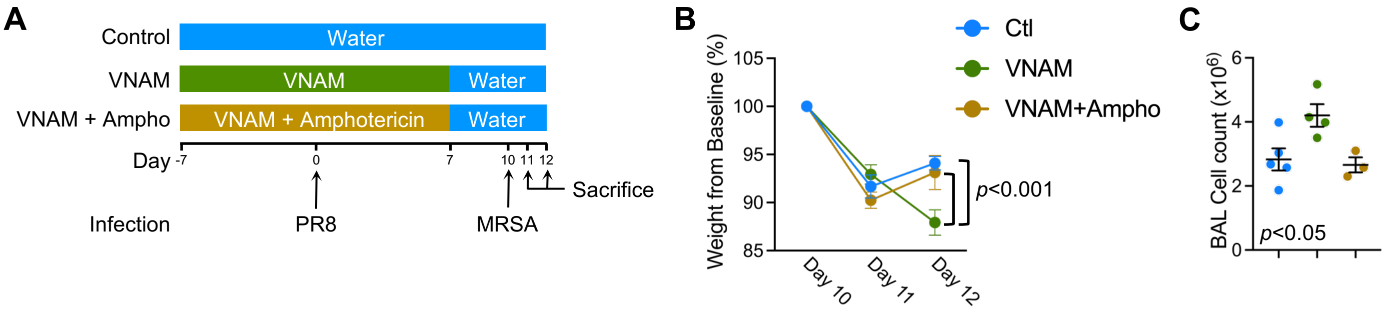

Supplemental Figure 11. Cotreatment with amphotericin reverses the worsened lung injury from the influenza-MRSA challenge in antibiotic-treated mice. A) Mice were infected with influenza (PR8, 250 PFU) at day 0 followed by MRSA at day 10. Control, antibiotics alone (VNAM), or VNAM and co-treatment with amphotericin (VNAM+Ampho) were started 7 days prior to PR8 infection to allow mice to equilibrate to the treatment and discontinued at day 7 to allow for it to wash out before MRSA challenge. **B)** Weight relative to that of day 10 showed a slower recovery after MRSA challenge in the VNAM-treated group compared to control and the VNAM-Ampho groups at day 11 and 12 (1 and 2 days after MRSA infection, respectively) by two-way ANOVA (n = 4-5). **C)** Mice in control, VNAM, and VNAM-Ampho groups were injured in the two-hit model and sacrificed on day 12 for evaluation of BAL total cell count. A one-way ANOVA was used to determine the *p* value (n = 3-5).

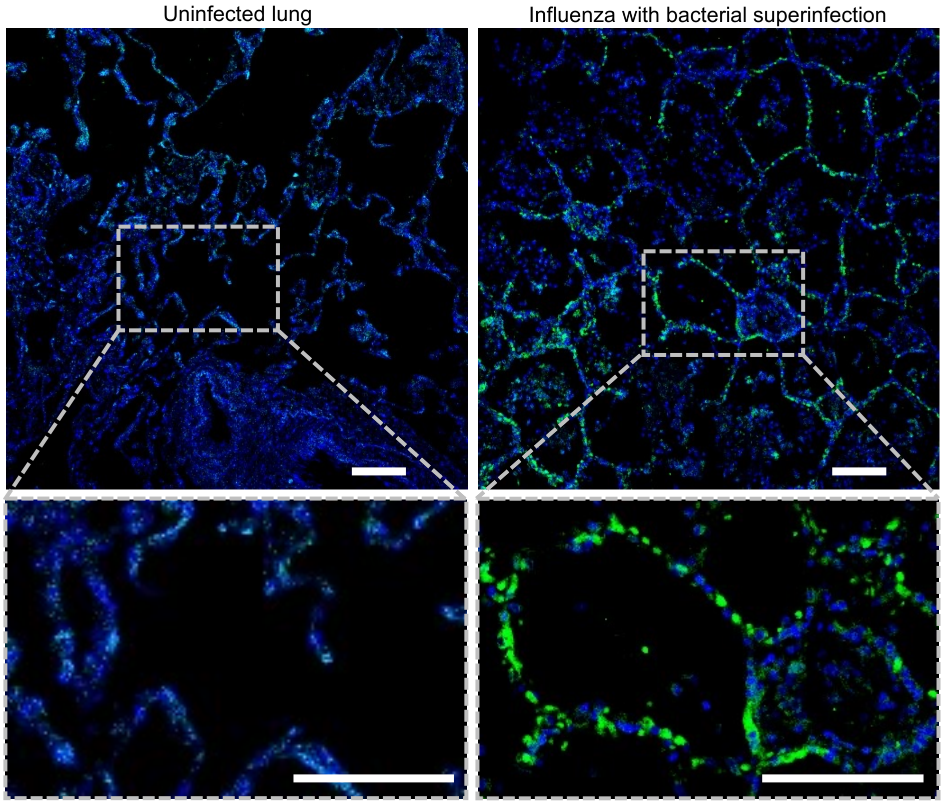

Supplemental Figure 12. Eosinophils are abundant in the lungs of a patient that died from influenza with *S. pneumoniae* superinfection. Lungs from an uninfected patient and one that succumbed to influenza followed by *S. pneumoniae* infection were immunostained for MBP-1 to identify eosinophils (green fluorescence) and counterstained with DAPI (blue). Scalebar = 100 μm.

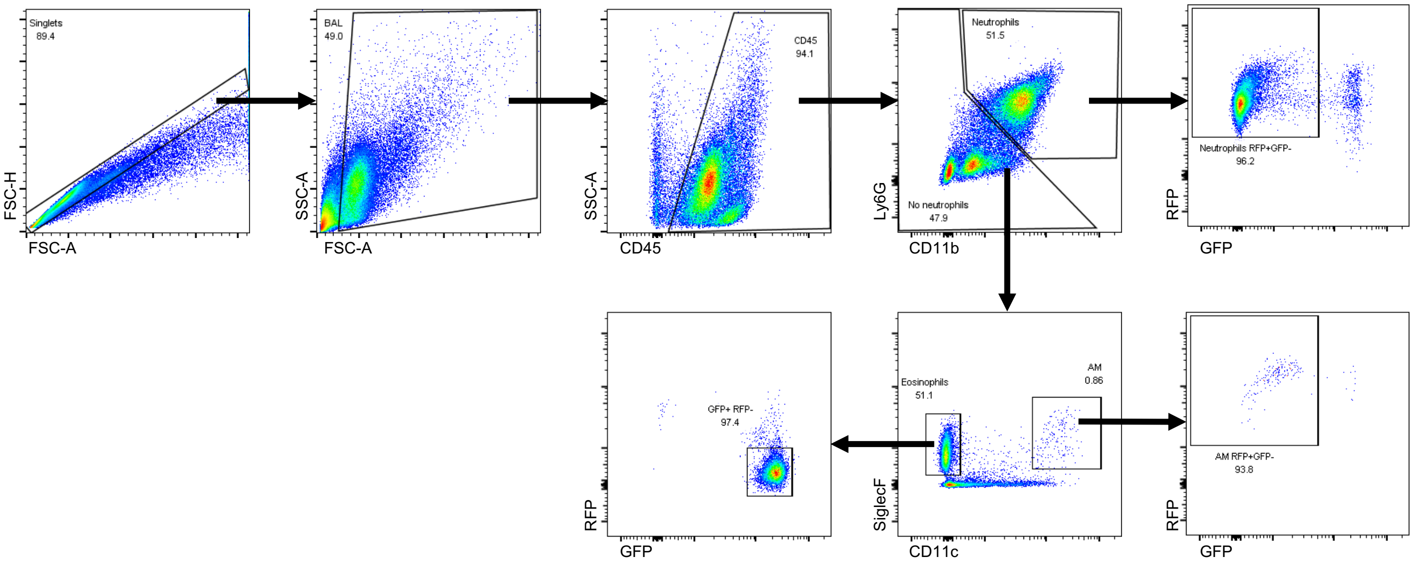

Supplemental Figure 13. Flow gating strategy for BAL immune cells in eoCre mice. BAL cells were immunostained for flow cytometry quantification of cell subsets. Eosinophils are the only population that are GFP^+ RFP^-.

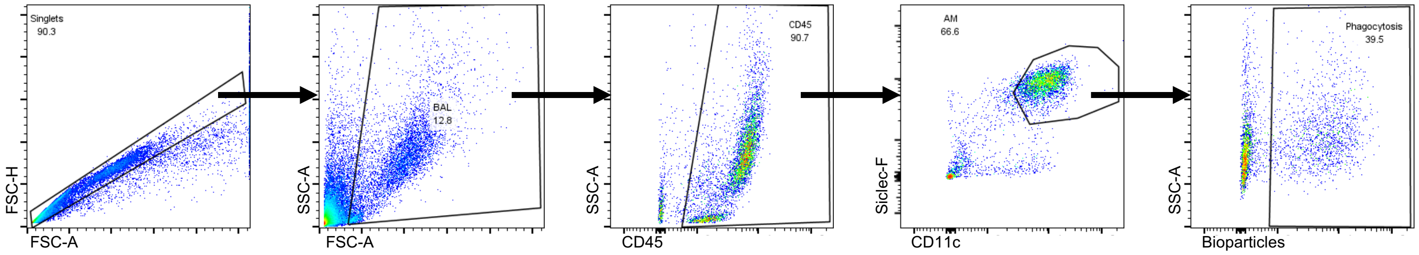

Supplemental Figure 14. Flow gating strategy for alveolar macrophage phagocytosis of MRSA bioparticles. MRSA-pHrhodo bioparticles were instilled into the lungs of mice. After 1 hour, mice were sacrificed, and the bronchoalveolar lavage was processed for flow cytometry. The percent of alveolar macrophages that have phagocytosed the bioparticle were determined.
